# Supplementary material for: Analysis of Log Data From an International Online Educational Assessment System: A Multi-State Survival Modeling Approach to Reaction Time Between and Across Action Sequence
Source: Psychometrika. 2025 Sep 1;90(4):1506–35. doi: 10.1017/psy.2025.10043 (PMC12660000; doi:10.1017/psy.2025.10043)
Supplement: Park et al. supplementary material [file S0033312325100434sup001.zip › Supplementary Material_notraceplot_v2_2025.pdf]

Supplementary Material: Analysis of log data from an international online educational assessment system: A multi-state survival modeling approach to reaction time between and across action sequence

1 Defined actions in CD Tally and Lamp Return test items

CD Tally

Action	Description	Freq (Correct)	Freq (Incorrect)
break	Break off	0	3
book_a	Add bookmark	30	26
book_a_c	Cancel to add bookmark	387	282
book_a_ok	Confirming to add bookmark	139	102
book_d	Delete bookmark	1	3
book_m	Manage the bookmark	51	46
book_m_c	Cancel to manage the bookmark	19	14
book_m_l	Bookmark list	8	13
book_m_ok	Confirming to manage the bookmark	14	18
combobox	Select combobox value	6217	2696
help	Help button for website	80	55
help_a	Help button for how to answer	636	694
keypress	Press the keyboard	2199	960
save	Save button	58	52
sch	Search button	203	61
sch_b	Click the previous button in the search engine	192	44
sch_n	Click the next button in the search engine	572	120
sch_ok	Conclude the search engine	946	224
so	Click the sort engine through the data menu on the spreadsheet page	1688	150

so_1_0	Sort by default	62	11
so_1_1	Sort by first column (Title)	119	26
so_1_2	Sort by second column (Artist)	119	30
so_1_3	Sort by third column (Genre)	3194	175
so_1_4	Sort by fourth column (Release Date)	100	26
so_2_asc	Sorts the spreadsheets in ascending order	1701	122
so_2_desc	Sorts the spreadsheets in descending order	462	30
so_c	Cancel the sort engine	240	91
so_ok	Click "Ok" after setting sorting options	3324	212
split_h	Split pages horizontally	1009	513
split_v	Split pages vertically	1135	508
ss	Switch to spreadsheet page	14328	4029
ss_copy	Click the copy button on the spreadsheet page	48	40
ss_cut	Click the cut button on the spreadsheet page	14	20
ss_data	Click the data menu on the spreadsheet page	1276	134
ss_edit	Click the edit button on the spreadsheet page	706	148
ss_file	Click the file button on the spreadsheet page	780	255
ss_help	Click the help button for spreadsheet	332	197
ss_pst	Click the paste button on the spreadsheet page	51	27
ss_save	Click the save button on the spreadsheet page	122	107
ss_sch	Click the search engine on the spreadsheet page	629	168
ss_so	Click the sort engine on the spreadsheet page	2045	264
wb	Switch to the website page	15211	4317
wb_book	Click the bookmark button on the website page	236	193
wb_e	Click the edit button on the website page	319	299
wb_f	Click the file menu on the website page	844	718
wb_h	Click the help button on the website page	321	337
wb_t_b	Click the back button on the website page toolbar	767	599
wb_t_book	Click the bookmark button on the website page toolbar	781	659
wb_t_f	Click the forward button on the website page toolbar	714	587
wb_t_h	Click the help button on the website page toolbar	1210	1012
wb_t_ho	Click the home button on the website page toolbar	973	748
wb_t_sch	Click the search engine on the website page toolbar	572	507

Table 1: The description and frequency of actions in CD Tally

Lamp Return

Action	Description	Freq (Correct)	Freq (Incorrect)
book	Click bookmark on the menu	21	69
break	Break off	0	3
copy	Click copy on the menu	148	295
edit	Click edit on the menu	349	705
em	Switch to Email page	10032	7955
em_add_f	Add the new folder on the email page	7	16
em_add_f_c	Cancel to add the new folder on the email page	2	9
em_add_f_ok	Confirming to add the new folder on the email page	10	2
em_cut	Click cut button on the email page	1	8
em_del_f	Delete the folder on the email page	1	1
em_del_f_c	Cancel to delete the folder on the email page	1	1
em_del_f_ok	Confirming to delete the folder on the email page	1	1
em_f_fold	Fold the folder on the email page	15	13
em_f_unfold	Unfold the folder on the email page	21	18
em_f_view	View the folder on the email page	2813	2454
em_for	Click forward email button on the email page	14	68
em_m_del	Click delete email button on the email page	3	7
em_m_drag	Drag email on the email page	113	167
em_m_drop	Drop email on the email page	11	38
em_m_move	Move email on the email page	34	53
em_m_view_0	View email 0 on the email page	1	7
em_m_view_101	View email 101 on the email page	340	316
em_m_view_102	View email 102 on the email page	315	281
em_m_view_103	View email 103 on the email page	293	280
em_m_view_104	View email 104 on the email page	377	356
em_m_view_105	View email 105 on the email page	352	342
em_m_view_106	View email 106 on the email page	338	326
em_m_view_107	View email 107 on the email page	376	367
em_m_view_108	View email 108 on the email page	382	369
em_m_view_109	View email 109 on the email page	470	465
em_m_view_110	View email 110 on the email page	400	365
em_m_view_111	View email 111 on the email page	469	452
em_m_view_112	View email 112 on the email page	500	497
em_m_view_113	View email 113 on the email page	447	440
em_m_view_114	View email 114 on the email page	347	345
em_m_view_115	View email 115 on the email page	1131	1196
em_m_view_116	View email 116 on the email page	630	601
em_m_view_117	View email 117 on the email page	608	583
em_m_view_118	View email 118 on the email page	546	545
em_m_view_119	View email 119 on the email page	1248	1290
em_m_view_120	View email 120 on the email page	652	665
em_m_view_305	View email 305 (confirm authorization number) on the email page	5253	2672

em_move	Click move menu on the email page	30	41
em_move_c	Cancel move menu on the email page	3	13
em_move_f	Click move folder on the email page	0	1
em_move_ok	Confirm move menu on the email page	23	15
em_new	Click new email button on the email page	90	323
em_re	Click reply email button on the email page	69	437
em_re_a	Click reply all email button on the email page	9	45
em_sch	Click search engine on the email page	62	48
em_sch_ok	Confirm the search engine on the email page	83	58
em_send	Click the send button on the email page	15	323
em_so	Click the sort engine on the email page	7	15
em_so_c	Cancel the sort engine on the email	0	5
em_so_desc	Sort the email in descending order on the email page	0	1
em_so_ok	Confirm the sort engine on the email page	7	10
em_trash	Click the trash email on the email page	6	9
file	Click the file menu	242	624
help	click the help button	103	370
keypress	Press the keyboard	10035	5916
message	Click the message button	32	213
paste	Click the paste button	132	288
splithorizontal	Split pages horizontally	74	152
splitvertical	Split pages vertically	65	115
url_ok	Confirm to move URL	76	473
view	Click view menu	17	48
wb	Switch to web page	10604	7635
wb_book_a	Add bookmark button on the web page	96	285
wb_book_a_c	Cancel to add a bookmark on the web page	49	147
wb_book_a_ok	Confirm to add a bookmark on the web page	18	85
wb_book_m	Click to manage bookmark button on the web page	2	12
wb_book_m_c	Cancel to manage bookmark on the web page	1	2
wb_book_m_ok	Confirming to manage the bookmark on the web page	0	3
wb_book_v	View the bookmark list on the web page	0	3
wb_hist_back	Click back button on the web page	12562	10408
wb_hist_for	Click forward button on the web page	355	527
wb_pg_0	Link to the main page of company on the web page	533	769
wb_pg_1	Link to Bedroom Lamps page on the web page	129	321
wb_pg_1_1	Link to a first sub-page on the Bedroom Lamps page	27	96
wb_pg_1_2	Link to a second sub-page on the Bedroom Lamps page	32	92
wb_pg_1_3	Link to a third sub-page on the Bedroom Lamps page	26	60
wb_pg_10	Link to About Us page on the web page	258	374
wb_pg_2	Link to the Desk Lamps page on the web page	2298	4405
wb_pg_2_1	Link to a first sub-page on the Desk Lamps page	832	2152
wb_pg_2_2	Link to a second sub-page on the Desk Lamps page	1154	2855
wb_pg_2_3	Link to a third sub-page on the Desk Lamps page	724	1853
wb_pg_3	Link to the Floor Lamps page on the web page	42	116

wb_pg_4	Link to the Table Lamps page on the web page	93	295
wb_pg_4_1	Link to a first sub-page on the Table Lamps page	26	108
wb_pg_4_2	Link to a second sub-page on the Table Lamps page	34	145
wb_pg_4_3	Link to a third sub-page on the Table Lamps page	22	115
wb_pg_5	Link to the New Arrivals page on the web page	75	147
wb_pg_5_1	Link to a first sub-page on the New Arrivals page	25	58
wb_pg_5_2	Link to a second sub-page on the New Arrivals page	21	47
wb_pg_6	Link to the SALE page on the web page	68	152
wb_pg_6_1	Link to a first sub-page on the SALE page	15	40
wb_pg_6_2	Link to a second sub-page on the SALE page	8	25
wb_pg_6_3	Link to a third sub-page on the SALE page	43	94
wb_pg_6_4	Link to a fourth sub-page on the SALE page	7	28
wb_pg_6_5	Link to a fifth sub-page on the SALE page	7	19
wb_pg_7	Link to Customer Comments page on the web page	927	1184
wb_pg_8	Link to Customer Service page	8400	8999
wb_pg_8_1	Link to Online questions page on the Customer Service page	832	1041
wb_pg_8_2	Link to view updated orders on the Customer Service page	1027	852
wb_pg_8_3	Link to obtain authorization number on the Customer Service page	5902	5573
wb_pg_8_3_1	Request authorization number on the Customer Service page	6177	6272
wb_pg_8_4	Link to view return form on the Customer Service page	5799	2829
wb_pg_8_4_reason_1	Select the first reason for return (Changed mind)	24	34
wb_pg_8_4_reason_2	Select the second reason for return (Item damaged or broken)	45	145
wb_pg_8_4_reason_3	Select the third reason for return (Don't like)	24	70
wb_pg_8_4_reason_4	Select the second reason for return (Wrong item shipped)	5014	1763
wb_pg_8_4_reason_5	Select the second reason for return (Poor quality)	7	17
wb_pg_8_4_reason_6	Select the second reason for return (Other)	88	312
wb_pg_8_4_request_0	Select a default request for returned items	2	1
wb_pg_8_4_request_1	Select a first request for returned items (Exchange for the correct item)	4975	2146
wb_pg_8_4_request_2	Select a second request for returned items (Order a different item)	39	123
wb_pg_8_4_request_3	Select a third request for returned items (Receive cash refund)	1	11
wb_pg_8_4_request_4	Select a fourth request for returned items (Receive credit refund)	0	3
wb_pg_8_4_request_5	Select a fifth request for returned items (Receive store credit)	1	3
wb_pg_8_4_submit	Submit the return form on the Customer Service page	5600	2139
wb_pg_9	Link to Employment Opportunities	73	93
wb_pg_pop1	Click the close button on pop-up system message 1	2898	7271
wb_pg_pop2	Click the close button on pop-up system message 2	6077	6003
wb_pg_pop3	Click the close button on pop-up system message 3	596	653
wb_pg_pop4	Click the close button on pop-up system message 4	4	22
wb_sch	Search button on the web page	26	134

Table 2: The description and frequency of actions in Lamp Return

2 Update step of MCMC

The MCMC at the iteration $t+1$ for the proposed model is given as follows:

1. Update the $\kappa_{0,m,l}$ using Gibbs sampler. For $m = 1, \cdots, E$ and $l = 1, \cdots, E$,

$$\pi\left(\kappa_{0,m,l} \mid \cdot\right) \sim \text{Gamma}\left(a_\kappa + \sum_{i=1}^{N}\sum_{j=1}^{E_i} I(a_{i,j-1} = m, a_{i,j} = l, c_i = 0), \frac{1}{1/b_\kappa + \sum_{i=1}^{N}\sum_{j=1}^{E_i} \delta_{m,l,i,j}^{(0)}}\right)$$

where, $\delta_{mlij}^{(0)} = \tau_i \cdot I(a_{i,j-1} = m) \cdot (t_{i,j} - t_{i,j-1}) \cdot \exp\left\{\sum_{p=1}^{P} \alpha_p x_{i,p} + \beta_{0,1} I(m \in \boldsymbol{A}) + \beta_{0,2} I(l \in \boldsymbol{A})\right\}$, and $a_\kappa = b_\kappa = 1.0$.

2. Update the $\kappa_{1,m,l}$ using Gibbs sampler. For $m = 1, \cdots, E$ and $l = 1, \cdots, E$,

$$\pi\left(\kappa_{1,m,l} \mid \cdot\right) \sim \text{Gamma}\left(a_\kappa + \sum_{i=1}^{N}\sum_{j=1}^{E_i} I(a_{i,j-1} = m, a_{i,j} = l, c_i = 1), \frac{1}{1/b_\kappa + \sum_{i=1}^{N}\sum_{j=1}^{E_i} \delta_{m,l,i,j}^{(1)}}\right)$$

where, $\delta_{mlij}^{(1)} = \tau_i \cdot I(a_{i,j-1} = m) \cdot (t_{i,j} - t_{i,j-1}) \cdot \exp\left\{\sum_{p=1}^{P} \alpha_p x_{i,p} + \beta_{1,1} I(m \in \boldsymbol{A}) + \beta_{1,2} I(l \in \boldsymbol{A})\right\}$, and $a_\kappa = b_\kappa = 1.0$.

3. Update the τ_i using Gibbs sampler. For $i = 1, \cdots, N$,

$$\pi\left(\tau_i \mid \cdot\right) \sim \text{Gamma}\left(a_\tau + E_i, \frac{1}{1/b_\tau + \sum_{j=1}^{E_i}\sum_{l=1}^{E} \delta_{mlij}^{(2)}}\right)$$

where, $\delta_{mlij}^{(2)} = \kappa_{c_i, a_{i,j-1}, l} \cdot (t_{i,j} - t_{i,j-1}) \cdot \exp\left\{\sum_{p=1}^{P} \alpha_p x_{i,p} + \beta_{c_i,1} I(a_{i,j-1} \in \boldsymbol{A}) + \beta_{c_i,2} I(l \in \boldsymbol{A})\right\}$, and $a_\tau = b_\tau = 1.0$.

4. Propose α_p^* from $\text{Normal}\left(\alpha_p^{(t)}, \sigma_{\alpha_p}^2\right)$ and accept the proposal with following probability. For $p = 1, \cdots, 5$,

$$r\left(\alpha_p^*, \alpha_p^{(t)}\right) = \frac{\pi\left(\alpha_p^*\right)}{\pi\left(\alpha_p^{(t)}\right)} \frac{P\left(\mathbf{Y} \mid \alpha_p^*, \boldsymbol{\Theta}_{-\alpha_p}^n\right)}{P\left(\mathbf{Y} \mid \alpha_p^{(t)}, \boldsymbol{\Theta}_{-\alpha_p}^n\right)} \frac{q\left(\alpha_p^* \to \alpha_p^{(t)}\right)}{q\left(\alpha_p^{(t)} \to \alpha_p^*\right)}.$$

where $\boldsymbol{\Theta}_{-\alpha_p}^n$ is the parameter set except α_p, $\sigma_{\alpha_1} = \sigma_{\alpha_3} = 0.03$, $\sigma_{\alpha_2} = \sigma_{\alpha_4} = 0.01$, and $\sigma_{\alpha_5} = 0.07$.

5. Propose the $\beta_{0,k}^*$ from $\text{Normal}\left(\beta_{0,k}^{(t)}, \sigma_{\beta_0}^2\right)$ and accept the proposal with probability. For $k = 1, 2$,

$$r\left(\beta_{0,k}^*, \beta_{0,k}^{(t)}\right) = \frac{\pi\left(\beta_{0,k}^*\right)}{\pi\left(\beta_{0,k}^{(t)}\right)} \frac{P\left(\mathbf{Y} \mid \beta_{0,k}^*, \boldsymbol{\Theta}_{-\beta_{0,k}}^n\right)}{P\left(\mathbf{Y} \mid \beta_{0,k}^{(t)}, \boldsymbol{\Theta}_{-\beta_{0,k}}^n\right)} \frac{q\left(\beta_{0,k}^* \to \beta_{0,k}^{(t)}\right)}{q\left(\beta_{0,k}^{(t)} \to \beta_{0,k}^*\right)}.$$

where $\boldsymbol{\Theta}_{-\beta_{0,k}}^n$ is the parameter set except $\beta_{0,k}$, and $\sigma_{\beta_0} = 0.2$.

6. Propose the $\beta^*_{1,k}$ from Normal$\left(\beta^{(t)}_{1,k}, \sigma^2_{\beta_1}\right)$ and accept the proposal with probability. For $k = 1, 2$,

$$r\left(\beta^*_{1,k}, \beta^{(t)}_{1,k}\right) = \frac{\pi\left(\beta^*_{1,k}\right)}{\pi\left(\beta^{(t)}_{1,k}\right)} \frac{P\left(\mathbf{Y} \mid \beta^*_{1,k}, \mathbf{\Theta}^n_{-\beta_{1,k}}\right)}{P\left(\mathbf{Y} \mid \beta^{(t)}_{1,k}, \mathbf{\Theta}^n_{-\beta_{1,k}}\right)} \frac{q\left(\beta^*_{1,k} \to \beta^{(t)}_{1,k}\right)}{q\left(\beta^{(t)}_{1,k} \to \beta^*_{1,k}\right)}.$$

where $\mathbf{\Theta}^n_{-\beta_{1,k}}$ is the parameter set except $\beta_{1,k}$, and $\sigma_{\beta_1} = 0.15$.

3 Country abbreviations

Abbreviation	Country
AT	Austria
BE	Belgium
DE	Germany
DK	Denmark
EE	Estonia
FI	Finland
GB	United Kingdom (England & Northern Ireland)
IE	Ireland
KR	South Korea
NL	Netherlands
NO	Norway
PL	Poland
SK	Slovakia
US	United States

Table 3: Country Abbreviations and Full Names

4 Top 5 and bottom 5 of $\kappa_{1,m,l}$ and $\kappa_{0,m,l}$ for CD Tally and Lamp Return test item

CD Tally

		Top 5			Bottom 5		
		From	To	Value	From	To	Value
Austria	Correct ($\kappa_{1,m,l}$)	ss_sch	keypress	9.66	ss	help_a	0.01
		sch	keypress	9.10	ss	so_1_3	0.01
		wb_t_f	wb_t_b	8.32	so_ok	sch_ok	0.02
		ss_data	so	6.74	so_ok	ss_help	0.02
		wb	ss	6.61	ss	ss_save	0.02
	Incorrect ($\kappa_{0,m,l}$)	ss_sch	keypress	6.23	ss	so_1_3	0.02
		wb	ss	5.60	ss	ss_help	0.04
		wb_t_b	wb_t_f	5.10	ss	ss_data	0.04
		split_h	split_v	4.13	ss	help_a	0.05
		ss_data	so	3.77	ss	ss_save	0.06
Belgium	Correct ($\kappa_{1,m,l}$)	ss_sch	keypress	11.26	ss	help_a	0.01
		combobox	ss	6.89	ss	ss_help	0.02
		sch	keypress	6.61	ss	ss_save	0.02
		wb	ss	6.06	so_1_3	so_1_2	0.03
		split_h	split_v	5.55	wb	wb_e	0.03
	Incorrect ($\kappa_{0,m,l}$)	split_h	split_v	5.47	ss	help_a	0.05
		wb_t_f	wb_t_b	4.45	ss	ss_help	0.05
		wb	ss	4.12	ss	ss_save	0.05
		ss_sch	keypress	4.00	ss	ss_data	0.07
		wb_t_ho	wb_t_book	3.94	wb	wb_e	0.08
Germany	Correct ($\kappa_{1,m,l}$)	ss_sch	keypress	12.03	ss	help_a	0.01
		wb	ss	7.00	so_ok	split_h	0.01
		sch	keypress	6.72	so_ok	ss_sch	0.01
		so_c	wb	6.52	wb	wb_t_book	0.02
		combobox	ss	6.50	wb	wb_t_sch	0.02
	Incorrect ($\kappa_{0,m,l}$)	wb	ss	6.01	ss	ss_edit	0.06
		wb	combobox	5.35	ss	keypress	0.06
		split_h	split_v	4.90	ss	ss_data	0.07
		sch	keypress	4.42	ss	ss_sch	0.07
		wb_t_b	wb_t_f	4.35	ss	help_a	0.09

Table 4: Top 5 and Bottom 5 of $\kappa_{1,m,l}$ and $\kappa_{0,m,l}$ for CD Tally test item in the Austria, Belgium, and Germany.

		Top 5			Bottom 5		
		From	To	Value	From	To	Value
Denmark	Correct ($\kappa_{1,m,l}$)	ss_sch	keypress	13.47	ss	ss_save	0.02
		wb	ss	7.88	so_ok	ss_save	0.02
		combobox	ss	7.54	so_ok	ss_file	0.03
		sch	keypress	7.22	so_1_3	so_c	0.03
		ss_data	so	6.46	wb	wb_t_sch	0.04
	Incorrect ($\kappa_{0,m,l}$)	ss_sch	keypress	7.47	ss	ss_data	0.04
		wb_t_f	wb_t_b	6.25	ss	ss_help	0.05
		wb	ss	4.84	ss	ss_edit	0.05
		so_2_asc	so_ok	4.73	wb	wb_h	0.07
		wb	combobox	4.44	ss	ss_save	0.08
Estonia	Correct ($\kappa_{1,m,l}$)	ss_sch	keypress	10.81	ss	help_a	0.01
		sch	keypress	8.78	ss	ss_save	0.01
		ss_data	so	7.25	ss	ss_copy	0.02
		wb	ss	6.31	so_ok	ss_copy	0.02
		combobox	ss	5.84	wb	wb_t_book	0.03
	Incorrect ($\kappa_{0,m,l}$)	wb	ss	5.61	ss	ss_copy	0.03
		ss_data	so	4.36	ss	split_h	0.04
		so_2_asc	so_ok	4.03	ss	ss_save	0.06
		wb_t_book	book_a_c	3.44	ss	ss_file	0.07
		wb_t_f	wb_t_b	3.05	ss	help_a	0.07
Finland	Correct ($\kappa_{1,m,l}$)	ss_sch	keypress	12.19	ss	sch_ok	0.01
		sch	keypress	8.74	ss	ss_save	0.02
		wb	ss	7.47	wb	wb_t_sch	0.02
		wb_t_b	wb_t_f	6.19	so_ok	split_v	0.03
		combobox	ss	6.14	so_ok	ss_file	0.03
	Incorrect ($\kappa_{0,m,l}$)	wb_t_b	wb_t_f	7.34	ss	sch_ok	0.02
		ss_sch	keypress	7.01	ss	ss_data	0.05
		wb	ss	5.53	wb	wb_h	0.06
		wb_t_book	book_a_c	4.00	ss	ss_edit	0.06
		split_h	split_v	3.96	ss	ss_save	0.08

Table 5: Top 5 and Bottom 5 of $\kappa_{1,m,l}$ and $\kappa_{0,m,l}$ for CD Tally test item in the Denmark, Estonia, and Finland.

		Top 5			Bottom 5		
		From	To	Value	From	To	Value
United Kingdom	Correct ($\kappa_{1,m,l}$)	ss_sch	keypress	11.70	ss	ss_save	0.01
		sch	keypress	8.73	so_ok	ss_save	0.02
		combobox	ss	8.73	ss	help_a	0.02
		wb	ss	6.63	so_ok	ss_file	0.02
		wb_t_book	book_a_c	5.82	wb	wb_t_sch	0.03
	Incorrect ($\kappa_{0,m,l}$)	wb_t_book	book_a_c	5.79	ss	ss_save	0.03
		wb	ss	5.72	ss	ss_edit	0.04
		split_h	split_v	5.09	ss	ss_data	0.04
		ss_sch	keypress	4.87	wb	wb_t_f	0.05
		sch_b	sch_n	4.57	ss	help_a	0.06
Ireland	Correct ($\kappa_{1,m,l}$)	ss_sch	keypress	8.75	ss	ss_help	0.02
		sch	keypress	7.01	ss	ss_save	0.02
		split_h	split_v	5.87	so_ok	ss_edit	0.02
		combobox	ss	5.37	ss	help_a	0.02
		wb	ss	5.22	wb	wb_t_b	0.03
	Incorrect ($\kappa_{0,m,l}$)	ss_sch	keypress	5.02	ss	ss_edit	0.02
		wb	ss	4.61	ss	ss_save	0.02
		wb_t_book	book_a_c	4.17	ss	ss_help	0.05
		wb	combobox	3.61	ss	ss_sch	0.07
		ss_data	so	3.52	ss	split_v	0.07
South Korea	Correct ($\kappa_{1,m,l}$)	ss_sch	keypress	11.29	so_ok	ss_edit	0.01
		sch	keypress	10.82	ss	help_a	0.01
		ss_data	so	8.15	so_ok	split_h	0.02
		so_2_asc	so_ok	5.27	ss	ss_save	0.02
		combobox	ss	4.96	ss	split_h	0.03
	Incorrect ($\kappa_{0,m,l}$)	wb	ss	5.72	ss	split_v	0.04
		wb_t_f	wb_t_b	5.10	ss	ss_help	0.08
		wb_t_book	book_a_c	3.89	ss	help_a	0.08
		ss_sch	keypress	3.83	ss	ss_save	0.08
		ss_data	so	3.75	ss	split_h	0.09

Table 6: Top 5 and Bottom 5 of $\kappa_{1,m,l}$ and $\kappa_{0,m,l}$ for CD Tally test item in the United Kingdom, Ireland, and South Korea.

		Top 5			Bottom 5		
		From	To	Value	From	To	Value
Netherlands	Correct ($\kappa_{1,m,l}$)	sch	keypress	9.97	ss	ss_save	0.01
		ss_sch	keypress	9.88	ss	help_a	0.01
		ss_data	so	8.27	so_ok	ss_help	0.01
		wb	ss	7.21	ss	ss_help	0.02
		combobox	ss	6.85	so_ok	ss_edit	0.02
	Incorrect ($\kappa_{0,m,l}$)	ss_sch	keypress	7.68	ss	ss_save	0.02
		wb_t_f	wb_t_b	5.57	ss	ss_help	0.04
		wb	ss	5.01	wb	wb_book	0.06
		sch	keypress	4.58	wb	wb_h	0.06
		wb_t_b	wb_t_f	4.24	ss	keypress	0.06
Norway	Correct ($\kappa_{1,m,l}$)	ss_sch	keypress	12.89	ss	sch_ok	0.01
		wb	ss	8.67	ss	ss_save	0.01
		sch	keypress	8.10	ss	sch_n	0.01
		ss_edit	so	7.61	so_ok	sch_ok	0.01
		combobox	ss	7.24	so_ok	ss_file	0.01
	Incorrect ($\kappa_{0,m,l}$)	ss_sch	keypress	7.79	ss	sch_n	0.02
		split_h	split_v	7.54	ss	ss_save	0.02
		wb	ss	6.43	ss	ss_data	0.03
		wb_t_f	wb_t_b	5.28	ss	sch_ok	0.03
		wb_t_b	wb_t_f	4.86	ss	help_a	0.04
Poland	Correct ($\kappa_{1,m,l}$)	ss_sch	keypress	12.96	ss	ss_help	0.02
		sch	keypress	7.09	ss	ss_save	0.02
		ss_data	so	6.20	so_ok	sch_ok	0.03
		split_h	split_v	6.02	so_ok	split_v	0.03
		wb_t_b	wb_t_f	5.79	wb	wb_t_ho	0.04
	Incorrect ($\kappa_{0,m,l}$)	ss_sch	keypress	7.02	ss	split_v	0.03
		wb	ss	4.05	ss	ss_data	0.03
		split_h	split_v	3.95	ss	ss_save	0.05
		wb	combobox	3.80	ss	ss_help	0.05
		wb_t_book	book_a_c	3.28	ss	keypress	0.05

Table 7: Top 5 and Bottom 5 of $\kappa_{1,m,l}$ and $\kappa_{0,m,l}$ for CD Tally test item in the Netherlands, Norway, and Poland.

		Top 5			Bottom 5		
		From	To	Value	From	To	Value
Slovakia	Correct ($\kappa_{1,m,l}$)	ss_sch	keypress	6.80	ss	ss_copy	0.01
		combobox	ss	4.47	ss	ss_save	0.01
		wb	combobox	3.96	ss	so_1_1	0.01
		ss_data	so	3.43	ss	split_v	0.02
		sch	keypress	3.34	wb	wb_t_b	0.03
	Incorrect ($\kappa_{0,m,l}$)	ss_sch	keypress	4.03	ss	ss_help	0.02
		split_h	split_v	3.79	ss	so_1_1	0.02
		wb	combobox	3.40	ss	ss_copy	0.02
		wb_t_b	wb_t_f	3.08	ss	ss_file	0.03
		wb	ss	2.91	ss	ss_save	0.03

Table 8: Top 5 and Bottom 5 of $\kappa_{1,m,l}$ and $\kappa_{0,m,l}$ for CD Tally test item in the Slovakia.

Lamp Return

		Top 5			Bottom 5		
		From	To	Value	From	To	Value
Austria	Correct ($\kappa_{1,m,l}$)	wb_pg_8_2	wb_pg_8	36.46	wb_pg_pop2	wb_hist_for	0.02
		wb_pg_8_1	wb_pg_8	29.98	wb_pg_pop2	wb_pg_0	0.02
		wb_pg_8_3	wb_pg_8_3_1	10.39	wb_pg_2	wb_pg_0	0.02
		wb_pg_8_4_submit	wb_pg_pop3	8.02	wb_pg_2	edit	0.02
		paste	keypress	6.82	keypress	em_send	0.03
	Incorrect ($\kappa_{0,m,l}$)	wb_pg_8_1	wb_pg_8	35.45	wb_pg_pop1	wb_pg_6_1	0.02
		wb_pg_8_2	wb_pg_8	35.05	wb_pg_2	edit	0.02
		wb_pg_8_3	wb_pg_8_3_1	13.13	wb_pg_pop2	wb_hist_for	0.02
		wb_pg_8_4_submit	wb_pg_pop3	12.30	wb_pg_2	wb_pg_0	0.03
		paste	keypress	7.46	wb_pg_pop1	wb_pg_6_4	0.03
Belgium	Correct ($\kappa_{1,m,l}$)	wb_pg_8_2	wb_pg_8	55.58	wb_pg_pop2	wb_hist_for	0.02
		wb_pg_8_1	wb_pg_8	25.53	wb_pg_pop2	wb_pg_0	0.02
		paste	keypress	8.76	keypress	em_re	0.02
		wb_pg_8_3	wb_pg_8_3_1	6.30	keypress	em_sch_ok	0.02
		wb_pg_2_2	wb_pg_pop1	5.32	wb_hist_back	file	0.02
	Incorrect ($\kappa_{0,m,l}$)	wb_pg_8_2	wb_pg_8	53.07	wb_pg_2	wb_pg_0	0.02
		wb_pg_8_1	wb_pg_8	30.94	wb_pg_pop2	file	0.02
		wb_pg_8_4_submit	wb_pg_pop3	8.99	wb_pg_pop2	wb_pg_0	0.02
		paste	keypress	8.00	wb_pg_8_3_1	wb_hist_back	0.03
		wb_pg_8_3	wb_pg_8_3_1	7.49	wb_pg_pop2	wb_hist_for	0.03
Germany	Correct ($\kappa_{1,m,l}$)	wb_pg_8_2	wb_pg_8	36.62	wb_pg_8	wb_hist_for	0.02
		wb_pg_8_1	wb_pg_8	25.86	wb_pg_pop2	wb_hist_for	0.02
		wb_pg_8_3	wb_pg_8_3_1	9.42	keypress	wb_pg_8	0.02
		wb_pg_8_4_submit	wb_pg_pop3	8.71	wb_hist_back	wb_pg_4	0.02
		paste	keypress	6.68	wb_hist_back	wb_pg_6	0.02
	Incorrect ($\kappa_{0,m,l}$)	wb_pg_8_2	wb_pg_8	40.83	wb_pg_8	wb_pg_0	0.01
		wb_pg_8_1	wb_pg_8	37.79	wb_pg_8	wb_hist_for	0.01
		paste	keypress	14.81	wb_pg_8	file	0.01
		wb_pg_8_4_submit	wb_pg_pop3	12.94	wb_pg_pop2	wb_hist_for	0.02
		wb_pg_8_3	wb_pg_8_3_1	11.43	wb_pg_8_3_1	wb	0.02

Table 9: Top 5 and Bottom 5 of $\kappa_{1,m,l}$ and $\kappa_{0,m,l}$ for Lamp Return test item in the Austria, Belgium, and Germany.

		Top 5			Bottom 5		
		From	To	Value	From	To	Value
Denmark	Correct ($\kappa_{1,m,l}$)	wb_pg_8_2	wb_pg_8	54.38	wb_pg_8	keypress	0.01
		wb_pg_8_1	wb_pg_8	24.18	keypress	help	0.01
		wb_pg_8_3	wb_pg_8_3_1	8.87	keypress	wb_pg_8	0.01
		wb_pg_2_3	wb_pg_pop1	8.44	keypress	url_ok	0.01
		wb_pg_2_2	wb_pg_pop1	7.37	keypress	em_m_view_116	0.01
	Incorrect ($\kappa_{0,m,l}$)	wb_pg_8_2	wb_pg_8	63.02	wb_pg_8	keypress	0.01
		wb_pg_8_1	wb_pg_8	46.90	wb_hist_back	file	0.01
		paste	keypress	11.65	wb_hist_back	wb_pg_8_4_reason_4	0.01
		wb_pg_8_4_submit	wb_pg_pop3	10.27	wb_hist_back	wb_book_a	0.01
		wb_pg_8_3	wb_pg_8_3_1	8.75	wb_hist_back	wb_pg_8_4_request_1	0.01
Estonia	Correct ($\kappa_{1,m,l}$)	wb_pg_8_2	wb_pg_8	64.46	wb_pg_8	help	0.01
		wb_pg_8_1	wb_pg_8	29.93	wb_pg_8	wb_pg_0	0.01
		paste	keypress	14.47	wb_hist_back	edit	0.02
		wb_pg_8_3	wb_pg_8_3_1	8.10	wb_hist_back	splithorizontal	0.02
		wb_pg_2_2	wb_pg_pop1	7.50	wb_hist_back	file	0.02
	Incorrect ($\kappa_{0,m,l}$)	wb_pg_8_2	wb_pg_8	49.80	wb_pg_2	file	0.01
		wb_pg_8_1	wb_pg_8	28.46	wb_pg_pop1	wb_pg_1_2	0.02
		paste	keypress	24.97	wb_pg_pop1	splithorizontal	0.02
		wb_pg_8_4_submit	wb_pg_pop3	10.36	wb_pg_8	wb_pg_0	0.02
		wb_pg_8_3	wb_pg_8_3_1	10.01	em_m_view_115	em_m_view_110	0.03
Finland	Correct ($\kappa_{1,m,l}$)	wb_pg_8_2	wb_pg_8	73.56	wb_pg_7	edit	0.01
		wb_pg_8_1	wb_pg_8	22.62	wb_pg_8	wb_pg_0	0.01
		paste	keypress	13.13	wb_hist_back	edit	0.01
		wb_pg_2_3	wb_pg_pop1	10.44	wb_hist_back	url_ok	0.01
		wb_pg_2_1	wb_pg_pop1	9.63	wb_hist_back	wb_pg_8_4_request_1	0.01
	Incorrect ($\kappa_{0,m,l}$)	wb_pg_8_2	wb_pg_8	53.37	wb_pg_2	wb_pg_0	0.02
		paste	keypress	26.18	wb_pg_7	wb_pg_0	0.02
		wb_pg_8_1	wb_pg_8	17.66	wb_pg_pop1	wb_pg_4_3	0.02
		wb_pg_8_3	wb_pg_8_3_1	10.76	wb_pg_pop1	wb_pg_5_2	0.02
		wb_pg_8_4_submit	wb_pg_pop3	10.31	wb_pg_pop1	wb_pg_4_1	0.02

Table 10: Top 5 and Bottom 5 of $\kappa_{1,m,l}$ and $\kappa_{0,m,l}$ for Lamp Return test item in the Denmark, Estonia, and Finland.

		Top 5			Bottom 5		
		From	To	Value	From	To	Value
United Kingdom	Correct ($\kappa_{1,m,l}$)	wb_pg_8_2	wb_pg_8	49.88	keypress	em_m_view_115	0.02
		wb_pg_8_1	wb_pg_8	28.78	keypress	wb_pg_8_4_reason_6	0.02
		wb_pg_8_3	wb_pg_8_3_1	10.17	keypress	em_send	0.02
		wb_pg_2_1	wb_pg_pop1	7.32	keypress	em_m_view_116	0.02
		wb_pg_8_4_submit	wb_pg_pop3	7.11	keypress	url_ok	0.02
	Incorrect ($\kappa_{0,m,l}$)	b_pg_8_2	wb_pg_8	40.22	wb_pg_2	wb_hist_for	0.01
		wb_pg_8_1	wb_pg_8	34.21	wb_pg_pop2	wb_pg_0	0.01
		paste	keypress	30.83	wb_pg_8_3_1	wb_hist_back	0.01
		wb_pg_8_4_submit	wb_pg_pop3	15.01	wb_pg_8_3_1	wb	0.02
		wb_pg_8_3	wb_pg_8_3_1	12.70	wb_hist_back	splithorizontal	0.02
Ireland	Correct ($\kappa_{1,m,l}$)	wb_pg_8_2	wb_pg_8	27.65	wb_pg_8_3_1	wb	0.02
		wb_pg_8_1	wb_pg_8	22.59	wb_pg_pop2	wb_pg_0	0.03
		wb_pg_8_3	wb_pg_8_3_1	8.48	wb_pg_pop2	file	0.03
		paste	keypress	7.91	wb_pg_8_3_1	wb_hist_back	0.03
		wb_pg_8_4_submit	wb_pg_pop3	6.30	keypress	em_m_view_115	0.03
	Incorrect ($\kappa_{0,m,l}$)	wb_pg_8_2	wb_pg_8	23.08	wb_pg_2	wb_hist_for	0.01
		wb_pg_8_4_submit	wb_pg_pop3	13.92	wb_pg_pop2	wb_pg_0	0.02
		paste	keypress	12.81	wb_pg_pop1	wb_pg_4_3	0.03
		wb_pg_8_3	wb_pg_8_3_1	11.93	wb_pg_8_3_1	wb	0.03
		wb_pg_8_1	wb_pg_8	9.76	wb_pg_8_3_1	wb_hist_back	0.03
South Korea	Correct ($\kappa_{1,m,l}$)	wb_pg_8_2	wb_pg_8	72.49	wb_hist_back	edit	0.01
		wb_pg_8_1	wb_pg_8	15.23	wb_pg_8	keypress	0.01
		wb_pg_8_4_submit	wb_pg_pop3	11.11	wb_hist_back	splitvertical	0.02
		wb_pg_8_3	wb_pg_8_3_1	11.04	wb_hist_back	book	0.02
		wb_pg_2_1	wb_pg_pop1	9.19	wb_pg_2	wb_hist_for	0.02
	Incorrect ($\kappa_{0,m,l}$)	wb_pg_8_2	wb_pg_8	59.29	wb_hist_back	wb_pg_8_4_reason_4	0.01
		wb_pg_8_1	wb_pg_8	19.94	wb_hist_back	edit	0.01
		wb_pg_8_4_submit	wb_pg_pop3	14.95	wb_hist_back	splitvertical	0.01
		wb_pg_8_3	wb_pg_8_3_1	13.45	wb_pg_2	url_ok	0.01
		wb_pg_2_1	wb_pg_pop1	9.38	wb_pg_pop1	wb_pg_6_4	0.02

Table 11: Top 5 and Bottom 5 of $\kappa_{1,m,l}$ and $\kappa_{0,m,l}$ for Lamp Return test item in the United Kingdom, Ireland, and South Korea.

		Top 5			Bottom 5		
		From	To	Value	From	To	Value
Netherlands	Correct ($\kappa_{1,m,l}$)	wb_pg_8_2	wb_pg_8	70.78	keypress	help	0.01
		wb_pg_8_1	wb_pg_8	26.71	keypress	splitvertical	0.01
		paste	keypress	12.42	keypress	wb_pg_8_4_reason_6	0.01
		wb_pg_8_4_submit	wb_pg_pop3	7.26	keypress	splithorizontal	0.01
		wb_pg_8_3	wb_pg_8_3_1	7.22	wb_pg_pop2	wb_book_a	0.01
	Incorrect ($\kappa_{0,m,l}$)	wb_pg_8_2	wb_pg_8	47.78	wb_pg_2	wb_book_a	0.01
		wb_pg_8_1	wb_pg_8	37.90	wb_pg_8_3_1	wb_hist_back	0.01
		paste	keypress	24.05	wb_pg_2	wb_pg_0	0.02
		wb_pg_8_4_submit	wb_pg_pop3	12.99	wb_pg_pop2	wb_book_a	0.02
		wb_pg_8_3	wb_pg_8_3_1	10.82	wb_pg_pop2	wb_pg_0	0.02
Norway	Correct ($\kappa_{1,m,l}$)	wb_pg_8_2	wb_pg_8	59.80	wb_pg_pop2	wb_hist_for	0.01
		wb_pg_8_1	wb_pg_8	39.56	wb_pg_2	wb_pg_0	0.02
		wb_pg_8_3	wb_pg_8_3_1	12.88	keypress	url_ok	0.02
		em_move_ok	em_m_move	12.80	keypress	wb_pg_7	0.02
		wb_pg_2_3	wb_pg_pop1	8.16	keypress	help	0.02
	Incorrect ($\kappa_{0,m,l}$)	wb_pg_8_2	wb_pg_8	68.84	wb_pg_2	wb_pg_0	0.01
		wb_pg_8_1	wb_pg_8	48.12	wb_pg_pop1	wb_pg_6_2	0.01
		paste	keypress	20.92	wb_pg_pop2	wb_hist_for	0.02
		wb_pg_8_3	wb_pg_8_3_1	12.66	wb_pg_8_3_1	wb_hist_back	0.02
		wb_pg_8_4_submit	wb_pg_pop3	8.49	wb_hist_back	wb_pg_8_4_reason_4	0.02
Poland	Correct ($\kappa_{1,m,l}$)	wb_pg_8_2	wb_pg_8	50.93	wb_pg_2	file	0.02
		wb_pg_8_3	wb_pg_8_3_1	10.49	wb_pg_pop2	wb_pg_0	0.02
		wb_pg_8_1	wb_pg_8	10.29	wb_pg_pop2	wb_book_a	0.02
		paste	keypress	8.01	wb_pg_8	file	0.02
		wb_pg_8_4_submit	wb_pg_pop3	7.48	wb_pg_8	keypress	0.02
	Incorrect ($\kappa_{0,m,l}$)	wb_pg_8_2	wb_pg_8	35.84	wb_pg_8_3_1	wb	0.02
		wb_pg_8_1	wb_pg_8	24.76	wb_pg_2	file	0.02
		wb_pg_8_4_submit	wb_pg_pop3	14.86	wb_pg_pop1	keypress	0.02
		wb_pg_8_3	wb_pg_8_3_1	13.06	wb_pg_2	wb_pg_0	0.02
		paste	keypress	10.72	wb_pg_pop1	wb_pg_5_1	0.02

Table 12: Top 5 and Bottom 5 of $\kappa_{1,m,l}$ and $\kappa_{0,m,l}$ for Lamp Return test item in the Netherlands, Norway, and Poland.

		Top 5			Bottom 5		
		From	To	Value	From	To	Value
Slovakia	Correct ($\kappa_{1,m,l}$)	wb_pg_8_2	wb_pg_8	13.37	wb_pg_pop2	keypress	0.02
		wb_pg_8_3	wb_pg_8_3_1	10.57	keypress	wb_pg_8_4	0.03
		wb_pg_8_4_submit	wb_pg_pop3	7.46	keypress	em_sch_ok	0.03
		wb_pg_8_1	wb_pg_8	6.99	keypress	file	0.03
		em	em_m_view_305	6.58	keypress	em_send	0.03
	Incorrect ($\kappa_{0,m,l}$)	wb_pg_8_2	wb_pg_8	14.45	wb_pg_8_3_1	wb_hist_back	0.02
		wb_pg_8_3	wb_pg_8_3_1	12.08	wb_pg_pop1	wb_pg_5_2	0.03
		wb_pg_8_4_submit	wb_pg_pop3	10.30	keypress	wb_pg_8_4	0.03
		em_m_drop	em_m_move	8.99	keypress	em_sch_ok	0.03
		wb_pg_8_4_request_1	keypress	5.55	em_m_view_115	em_m_view_120	0.04

Table 13: Top 5 and Bottom 5 of $\kappa_{1,m,l}$ and $\kappa_{0,m,l}$ for Lamp Return test item in the Slovakia.

5 Heatmap and network visualization of difference in transition probability between correct and incorrect groups for CD Tally and Lamp Return test items

CD Tally

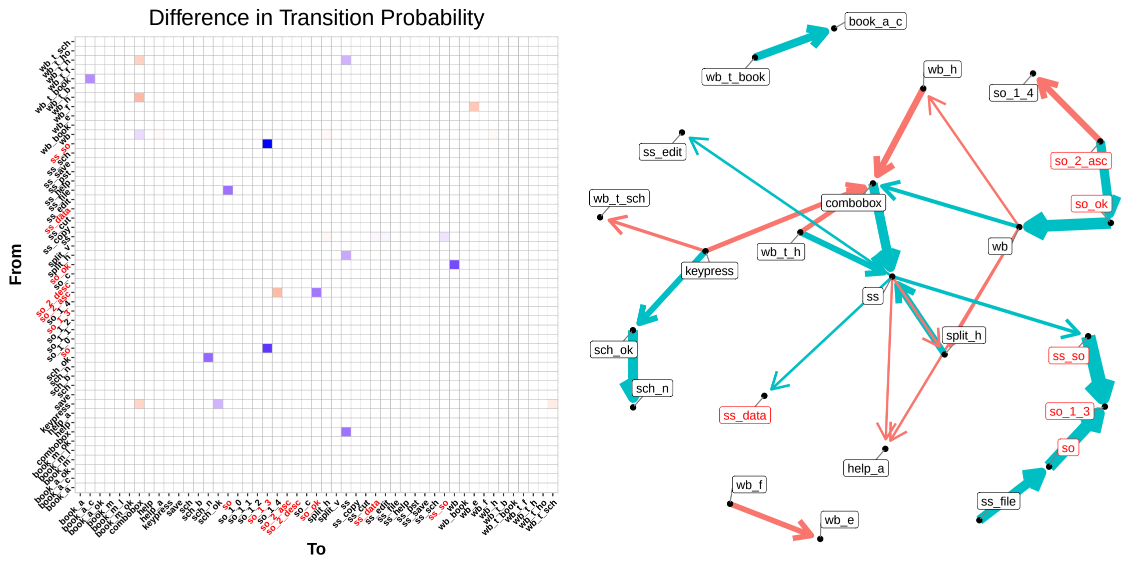

(a) Heatmap (b) Network visualization

Figure 1: The difference in transition probability between the correct and incorrect answer groups using (a) a heatmap and (b) a network visualization for CD Tally test item in the Austria.

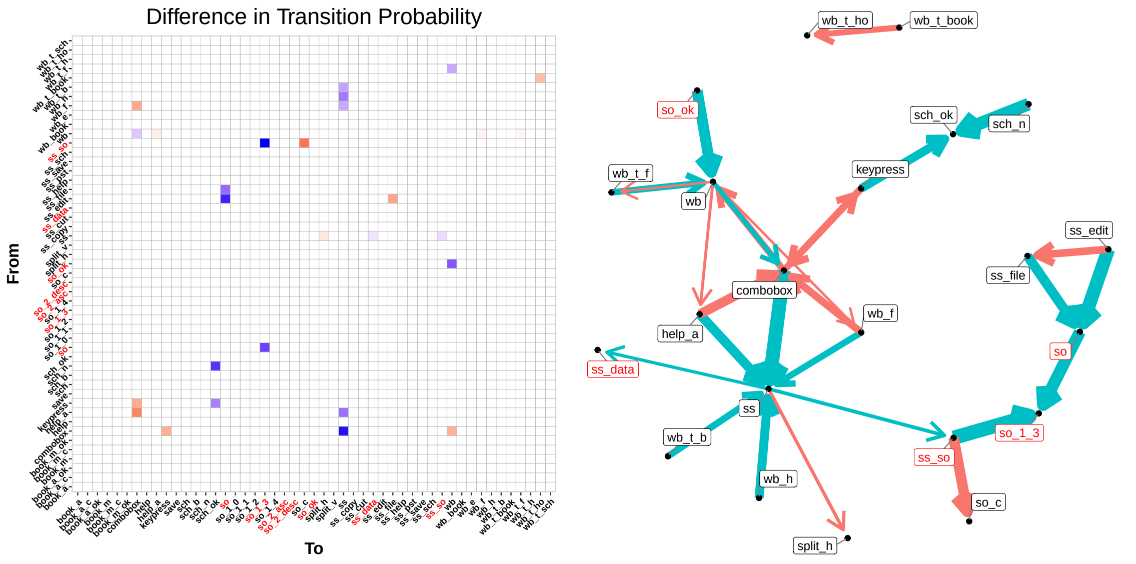

(a) Heatmap (b) Network visualization

Figure 2: The difference in transition probability between the correct and incorrect answer groups using (a) a heatmap and (b) a network visualization for CD Tally test item in the Belgium.

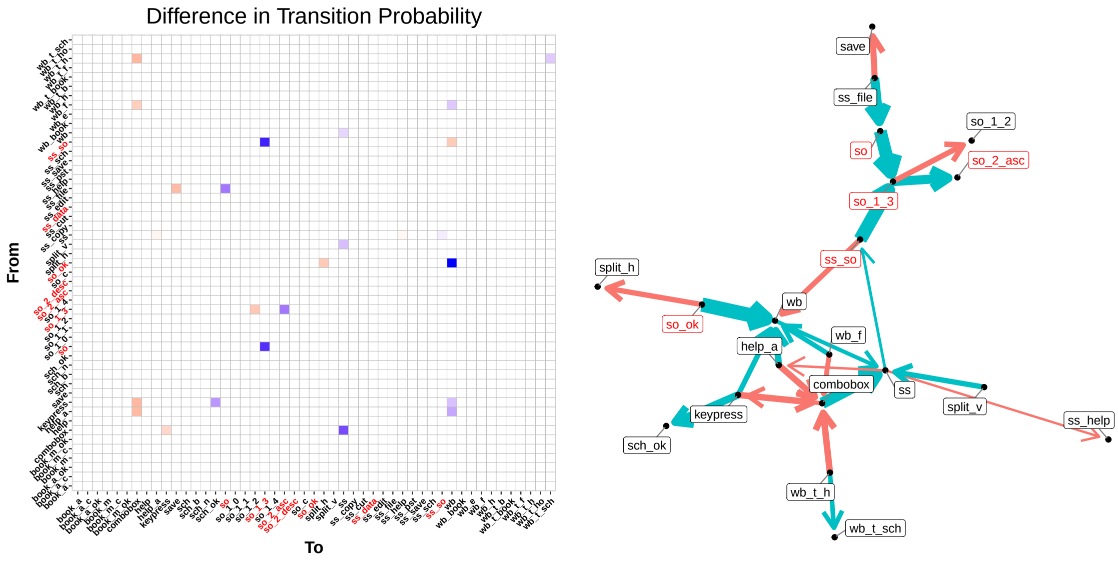

(a) Heatmap (b) Network visualization

Figure 3: The difference in transition probability between the correct and incorrect answer groups using (a) a heatmap and (b) a network visualization for CD Tally test item in the Germany.

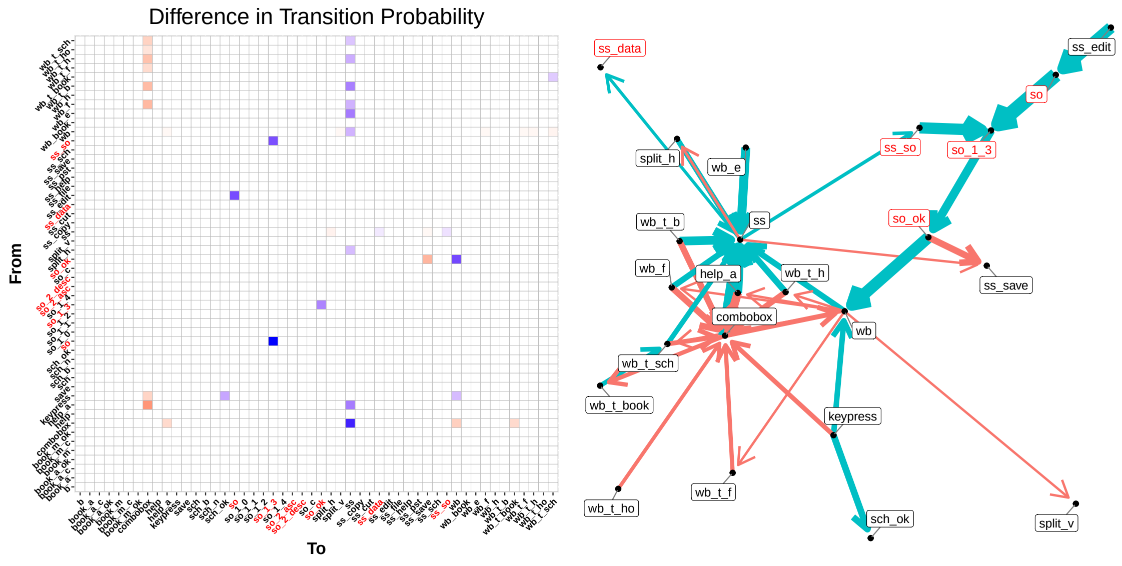

(a) Heatmap (b) Network visualization

Figure 4: The difference in transition probability between the correct and incorrect answer groups using (a) a heatmap and (b) a network visualization for CD Tally test item in the Denmark.

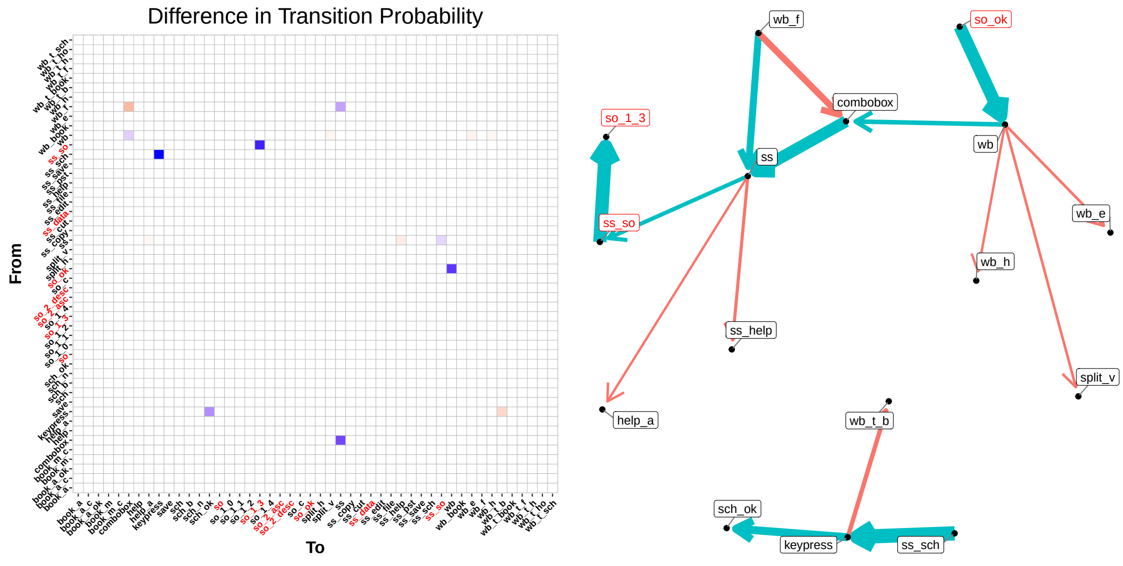

(a) Heatmap (b) Network visualization

Figure 5: The difference in transition probability between the correct and incorrect answer groups using (a) a heatmap and (b) a network visualization for CD Tally test item in the Estonia.

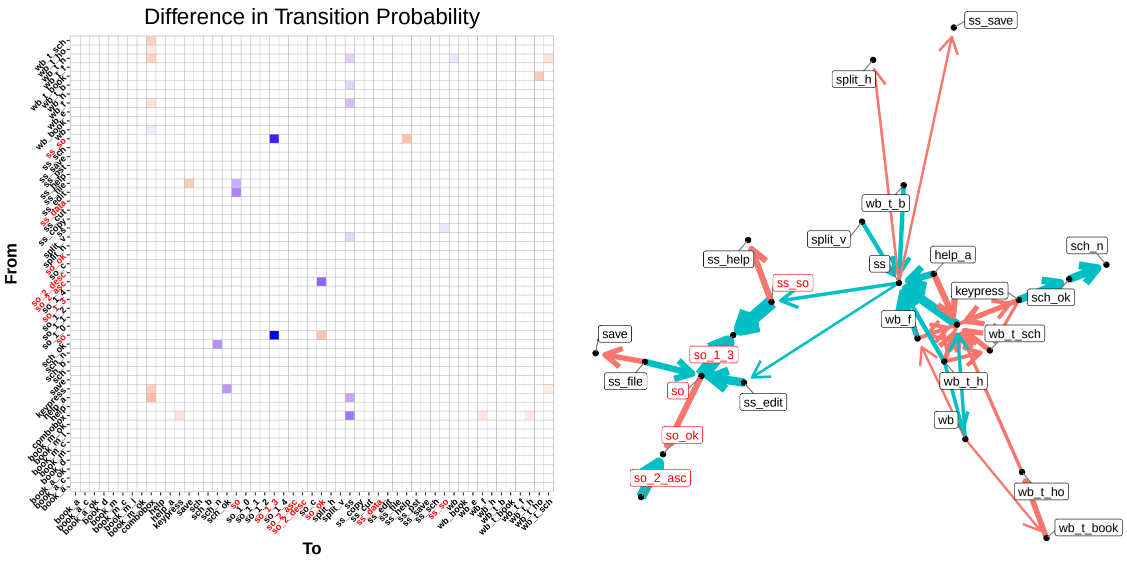

(a) Heatmap (b) Network visualization

Figure 6: The difference in transition probability between the correct and incorrect answer groups using (a) a heatmap and (b) a network visualization for CD Tally test item in the Finland.

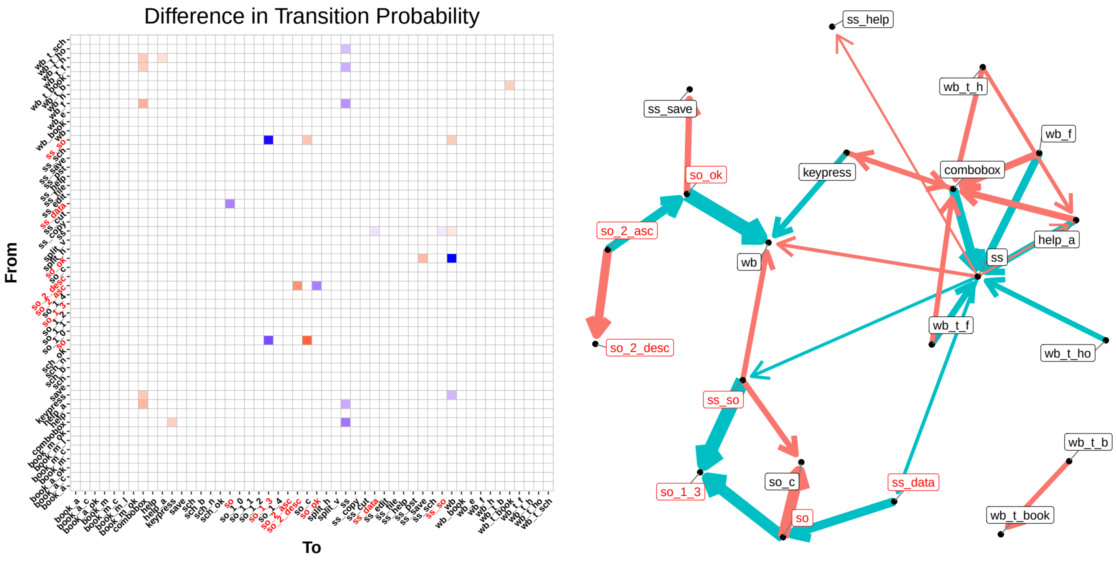

(a) Heatmap (b) Network visualization

Figure 7: The difference in transition probability between the correct and incorrect answer groups using (a) a heatmap and (b) a network visualization for CD Tally test item in the United Kingdom.

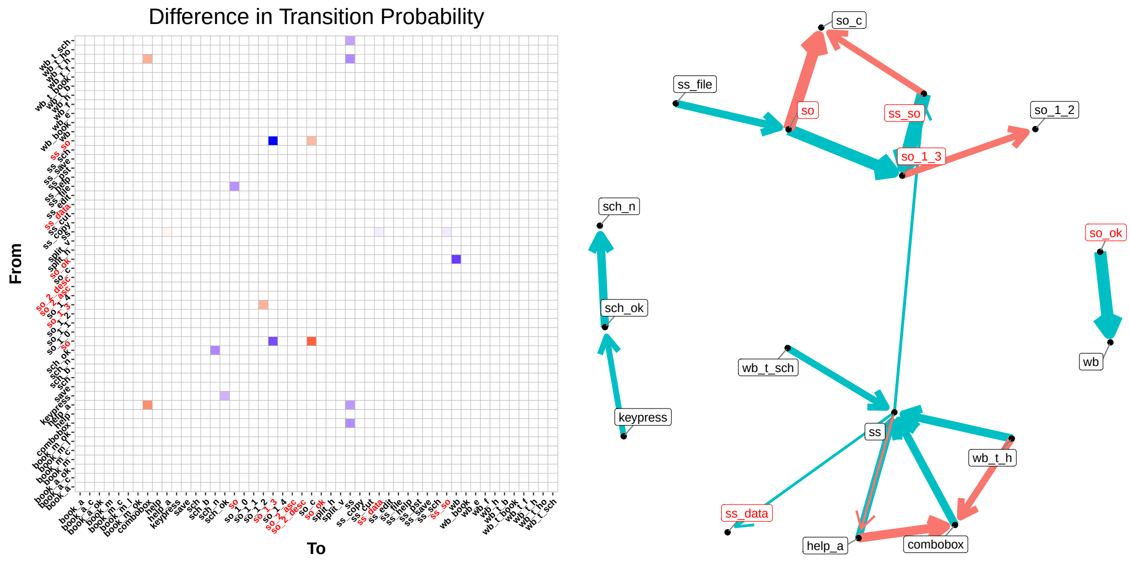

(a) Heatmap (b) Network visualization

Figure 8: The difference in transition probability between the correct and incorrect answer groups using (a) a heatmap and (b) a network visualization for CD Tally test item in the Ireland.

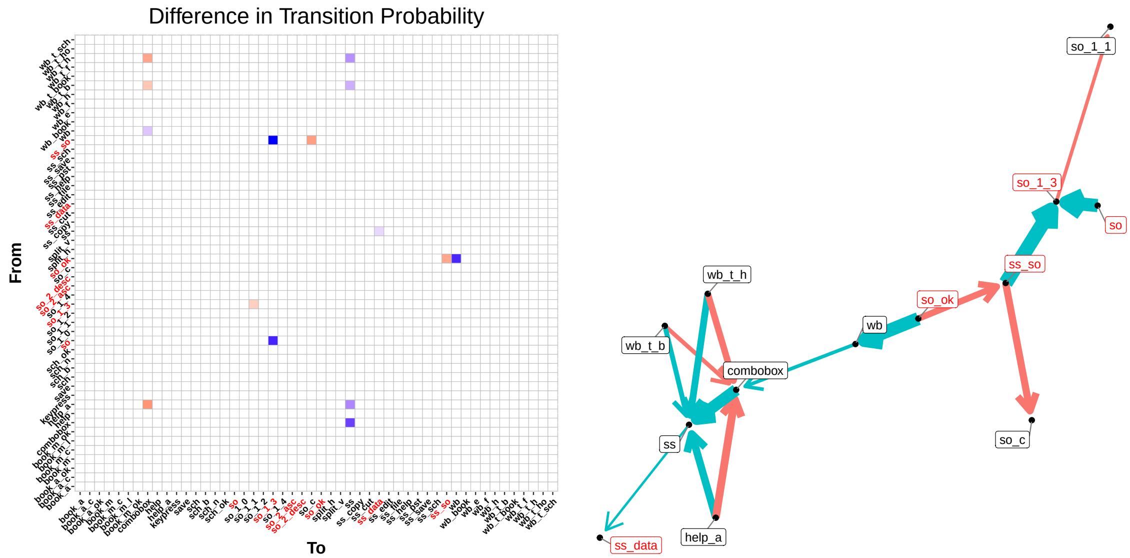

(a) Heatmap (b) Network visualization

Figure 9: The difference in transition probability between the correct and incorrect answer groups using (a) a heatmap and (b) a network visualization for CD Tally test item in the South Korea.

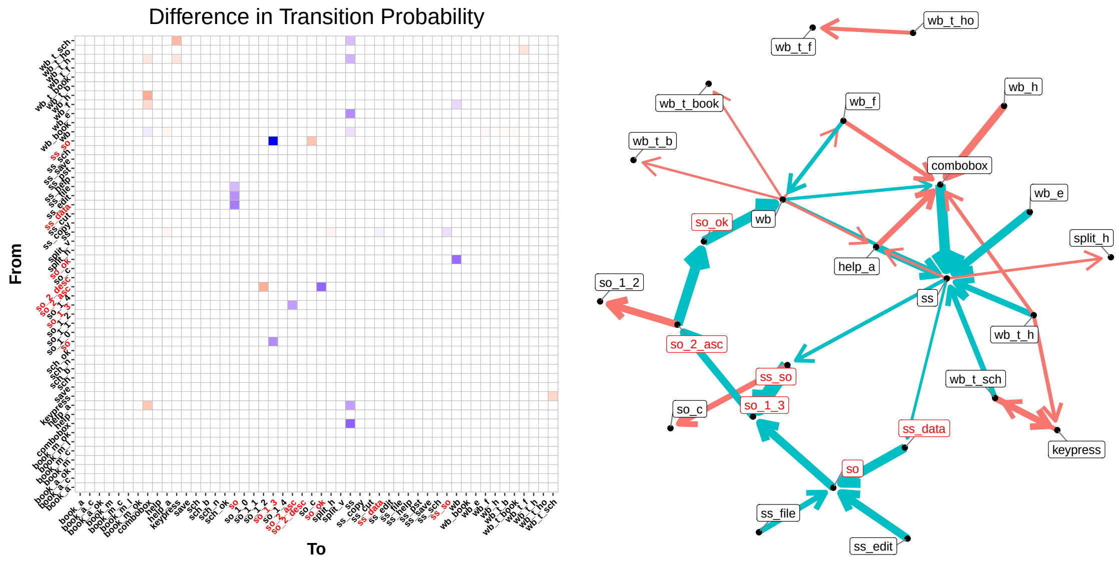

(a) Heatmap

(b) Network visualization

Figure 10: The difference in transition probability between the correct and incorrect answer groups using (a) a heatmap and (b) a network visualization for CD Tally test item in the Netherlands.

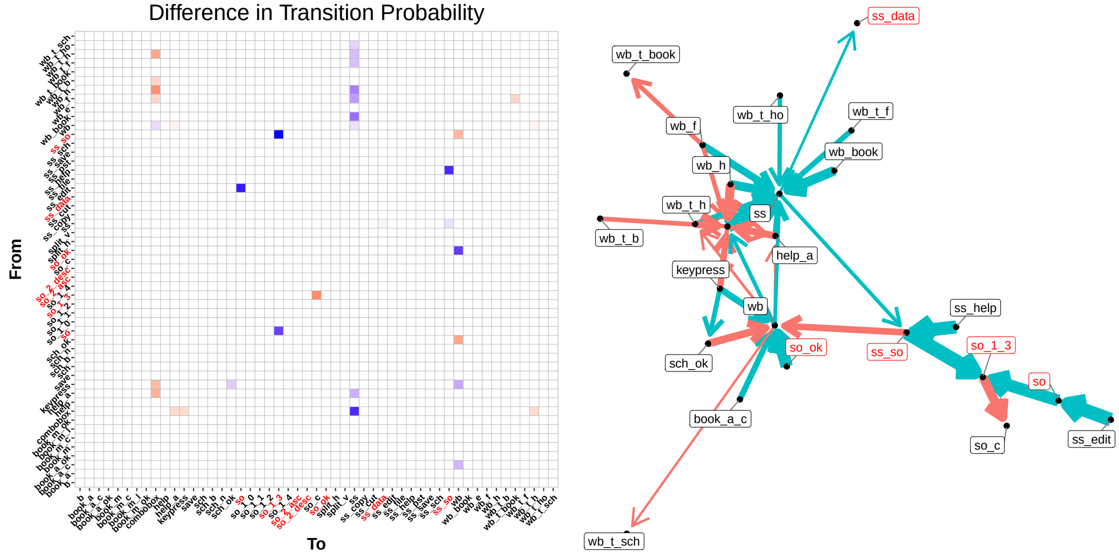

(a) Heatmap

(b) Network visualization

Figure 11: The difference in transition probability between the correct and incorrect answer groups using (a) a heatmap and (b) a network visualization for CD Tally test item in the Norway.

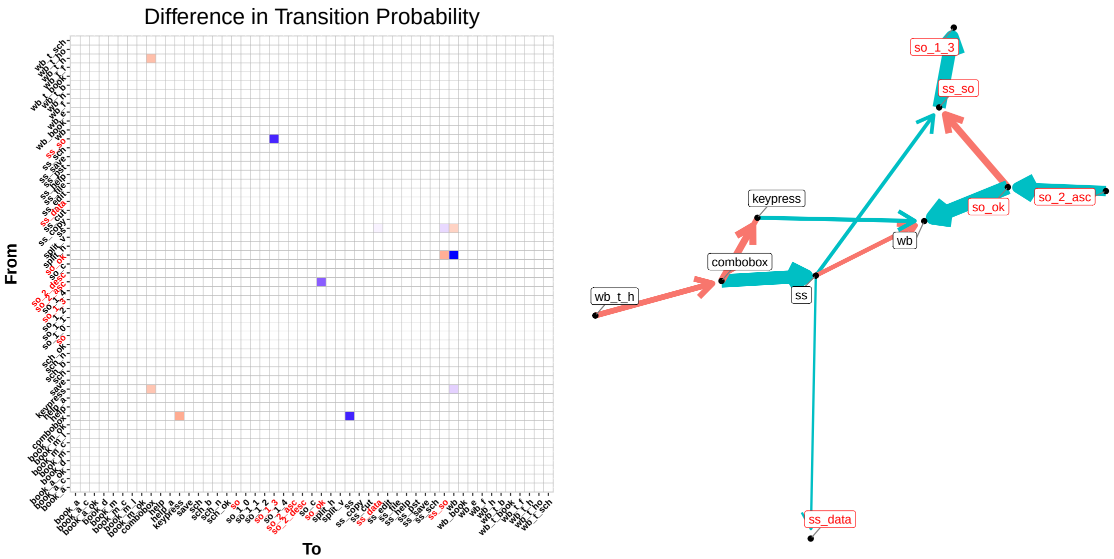

(a) Heatmap (b) Network visualization

Figure 12: The difference in transition probability between the correct and incorrect answer groups using (a) a heatmap and (b) a network visualization for CD Tally test item in the Poland.

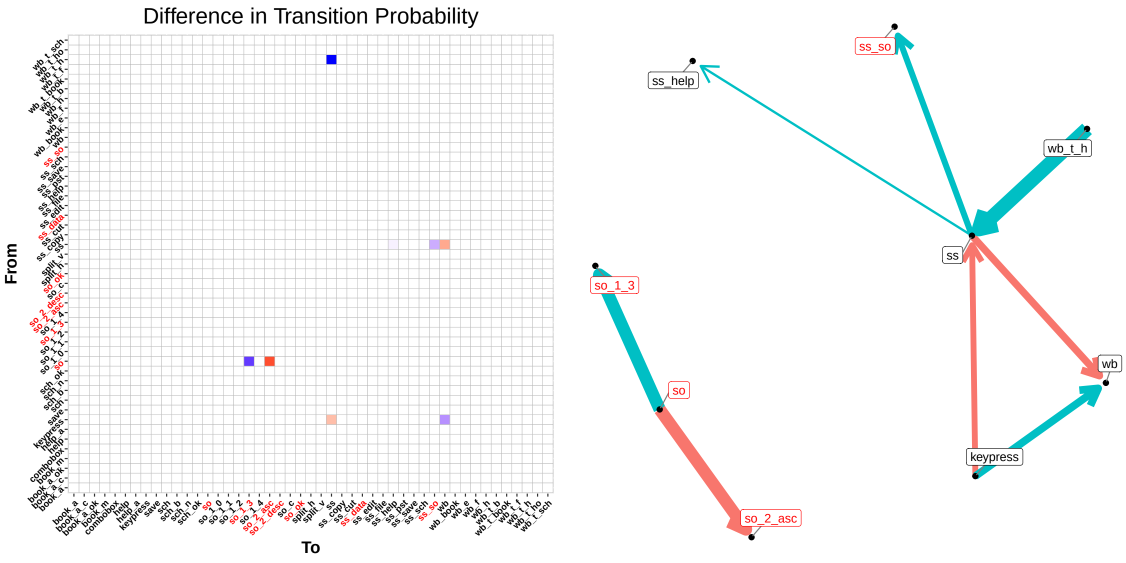

(a) Heatmap (b) Network visualization

Figure 13: The difference in transition probability between the correct and incorrect answer groups using (a) a heatmap and (b) a network visualization for CD Tally test item in the Slovakia.

Lamp Return

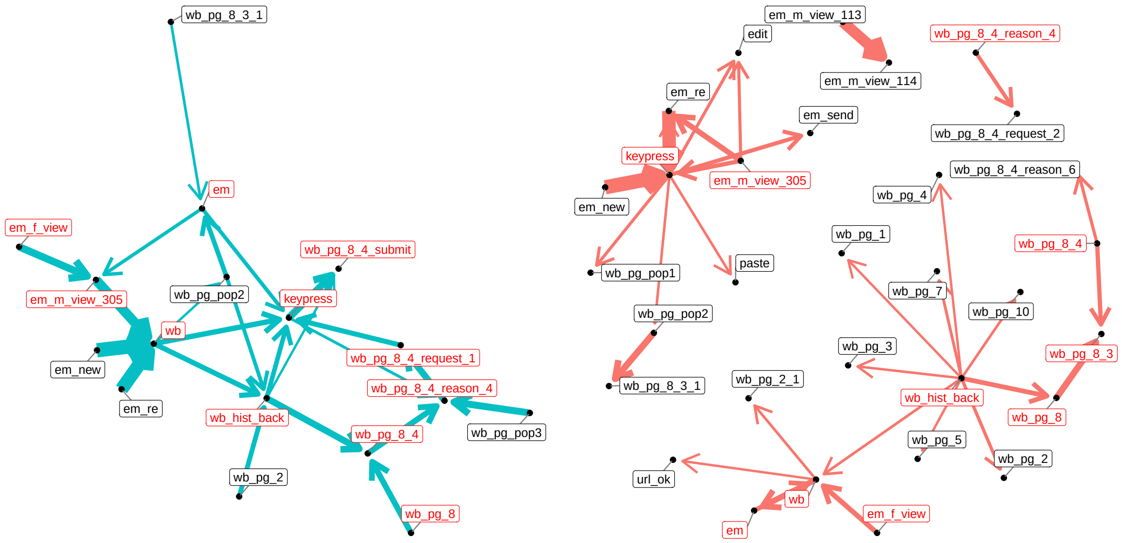

(a) (b)

Figure 14: Network visualization of transition probability differences for the Lamp Return test item in the Austria. (a) shows significantly higher transition probabilities for the correct group (blue arrows), while (b) shows higher probabilities for the incorrect group (red arrows).

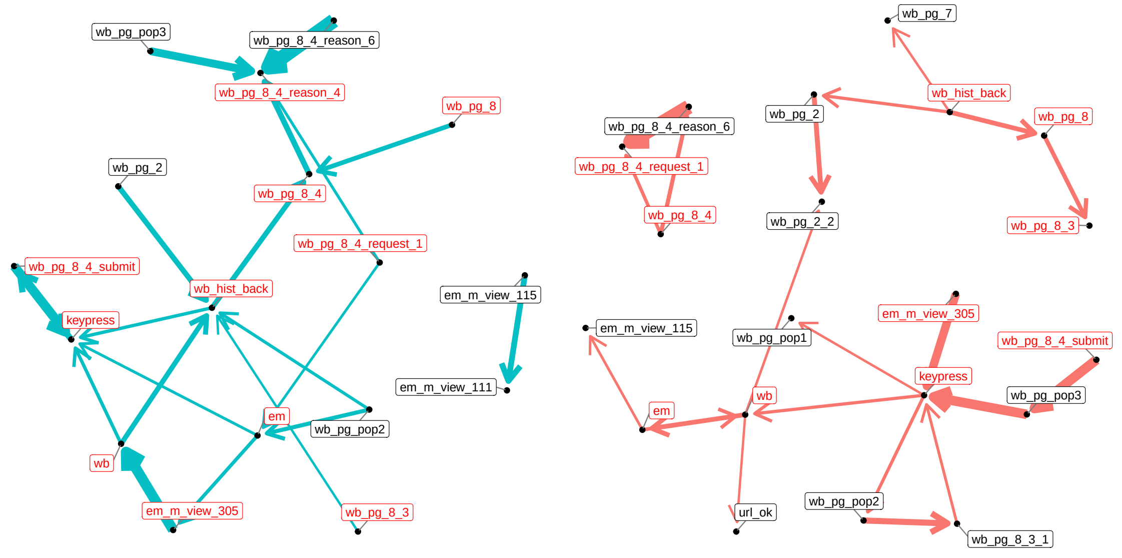

(a) (b)

Figure 15: Network visualization of transition probability differences for the Lamp Return test item in the Belgium. (a) shows significantly higher transition probabilities for the correct group (blue arrows), while (b) shows higher probabilities for the incorrect group (red arrows).

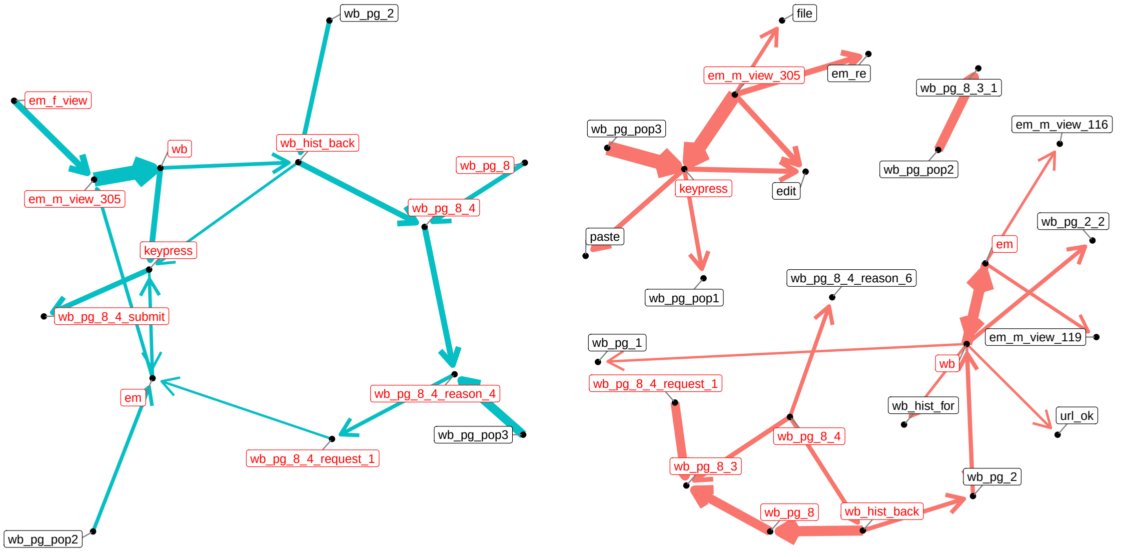

(a) (b)

Figure 16: Network visualization of transition probability differences for the Lamp Return test item in the Germany. (a) shows significantly higher transition probabilities for the correct group (blue arrows), while (b) shows higher probabilities for the incorrect group (red arrows).

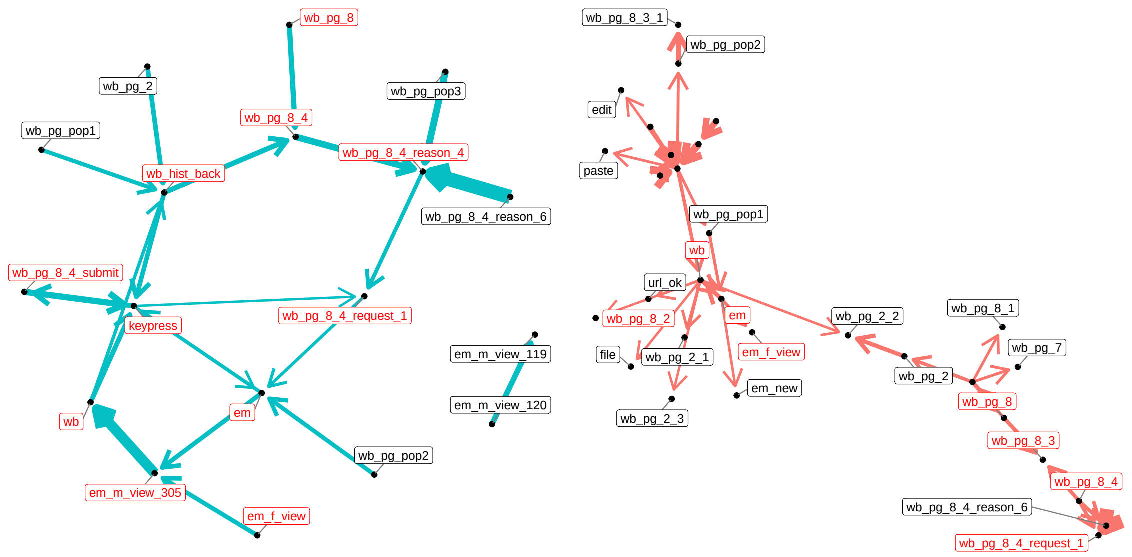

(a) (b)

Figure 17: Network visualization of transition probability differences for the Lamp Return test item in the Denmark. (a) shows significantly higher transition probabilities for the correct group (blue arrows), while (b) shows higher probabilities for the incorrect group (red arrows).

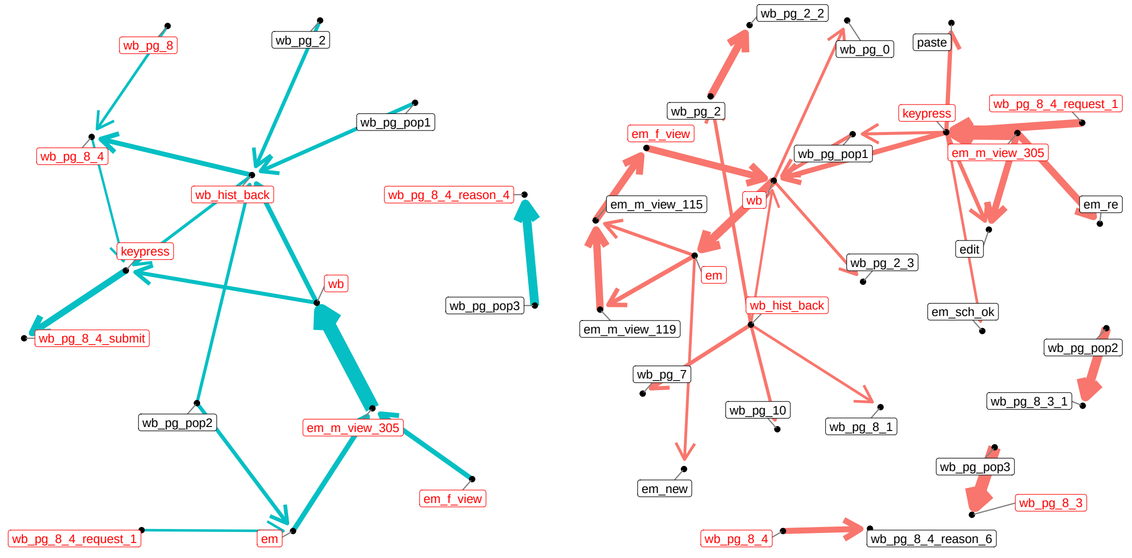

(a) (b)

Figure 18: Network visualization of transition probability differences for the Lamp Return test item in the Estonia. (a) shows significantly higher transition probabilities for the correct group (blue arrows), while (b) shows higher probabilities for the incorrect group (red arrows).

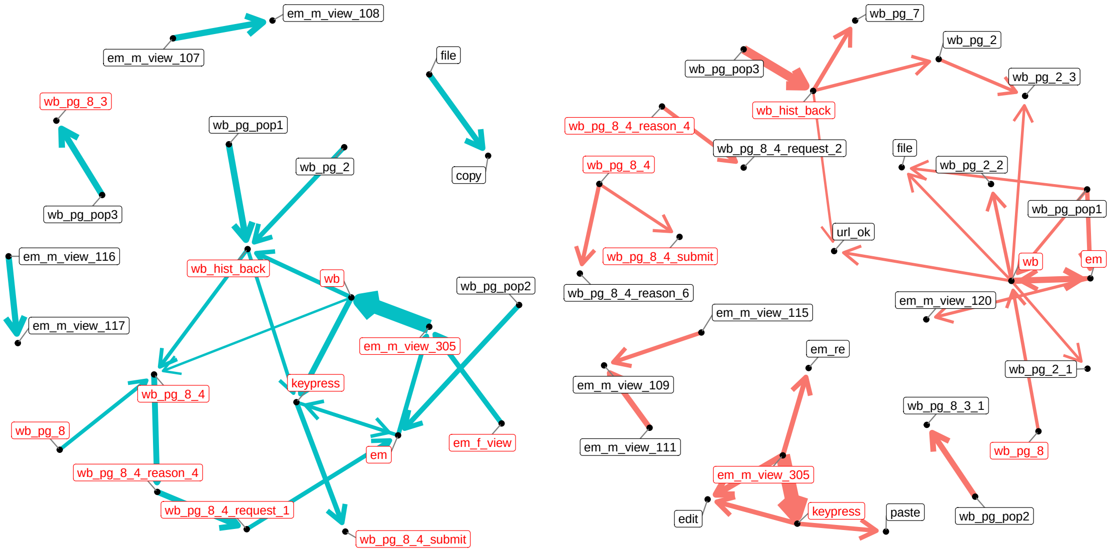

(a) (b)

Figure 19: Network visualization of transition probability differences for the Lamp Return test item in the Finland. (a) shows significantly higher transition probabilities for the correct group (blue arrows), while (b) shows higher probabilities for the incorrect group (red arrows).

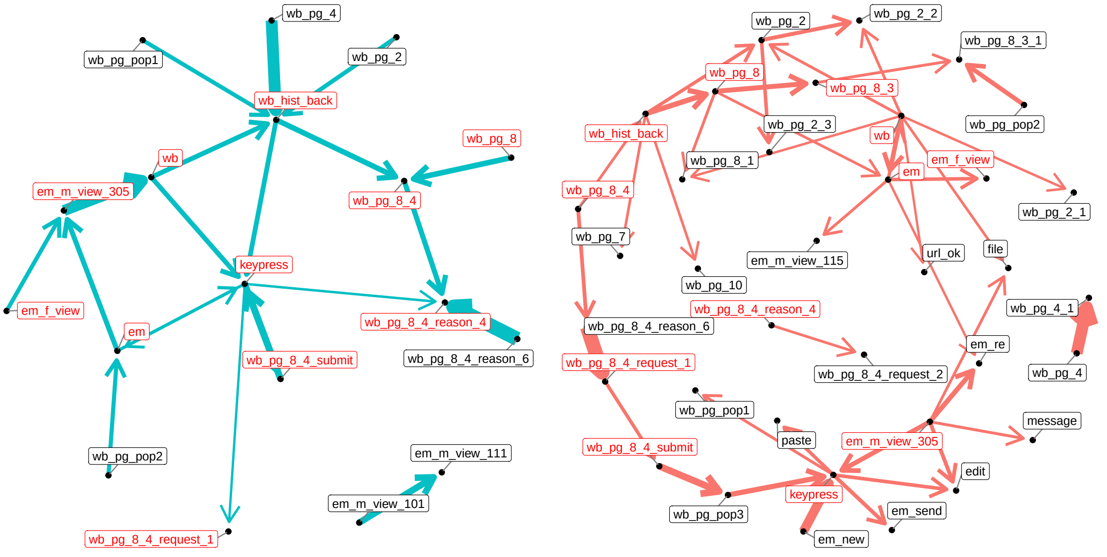

(a) (b)

Figure 20: Network visualization of transition probability differences for the Lamp Return test item in the United Kingdom. (a) shows significantly higher transition probabilities for the correct group (blue arrows), while (b) shows higher probabilities for the incorrect group (red arrows).

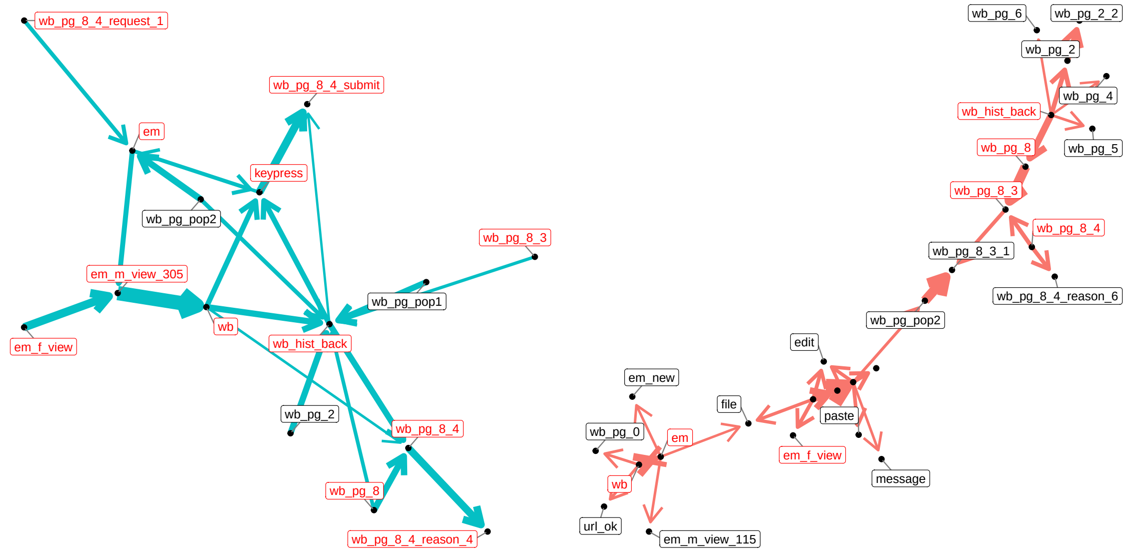

(a) (b)

Figure 21: Network visualization of transition probability differences for the Lamp Return test item in the Ireland. (a) shows significantly higher transition probabilities for the correct group (blue arrows), while (b) shows higher probabilities for the incorrect group (red arrows).

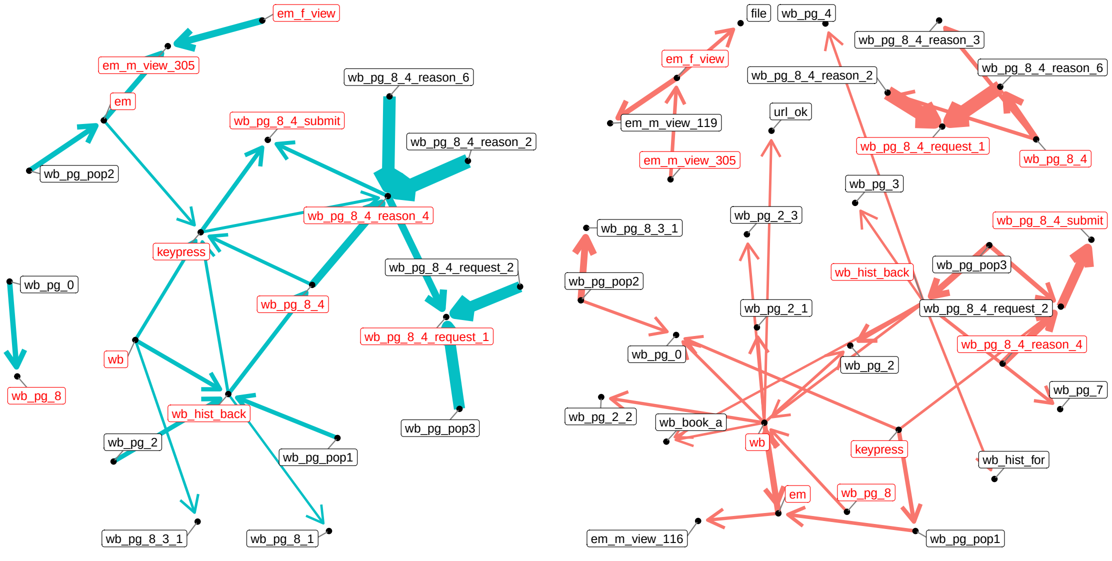

(a) (b)

Figure 22: Network visualization of transition probability differences for the Lamp Return test item in the South Korea. (a) shows significantly higher transition probabilities for the correct group (blue arrows), while (b) shows higher probabilities for the incorrect group (red arrows).

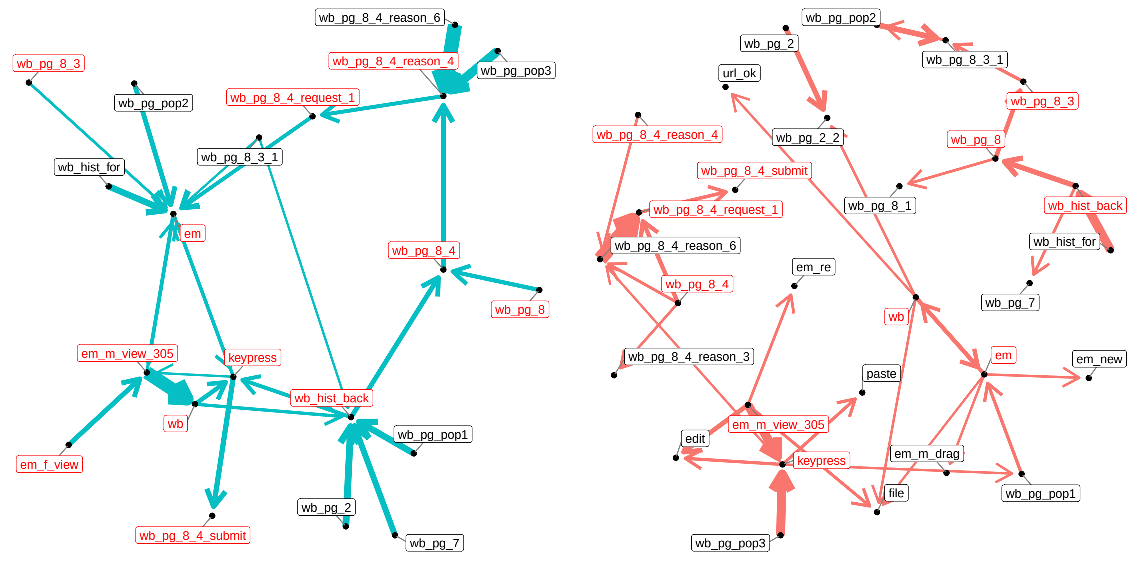

(a) (b)

Figure 23: Network visualization of transition probability differences for the Lamp Return test item in the Estonia. (a) shows significantly higher Netherlands probabilities for the correct group (blue arrows), while (b) shows higher probabilities for the incorrect group (red arrows).

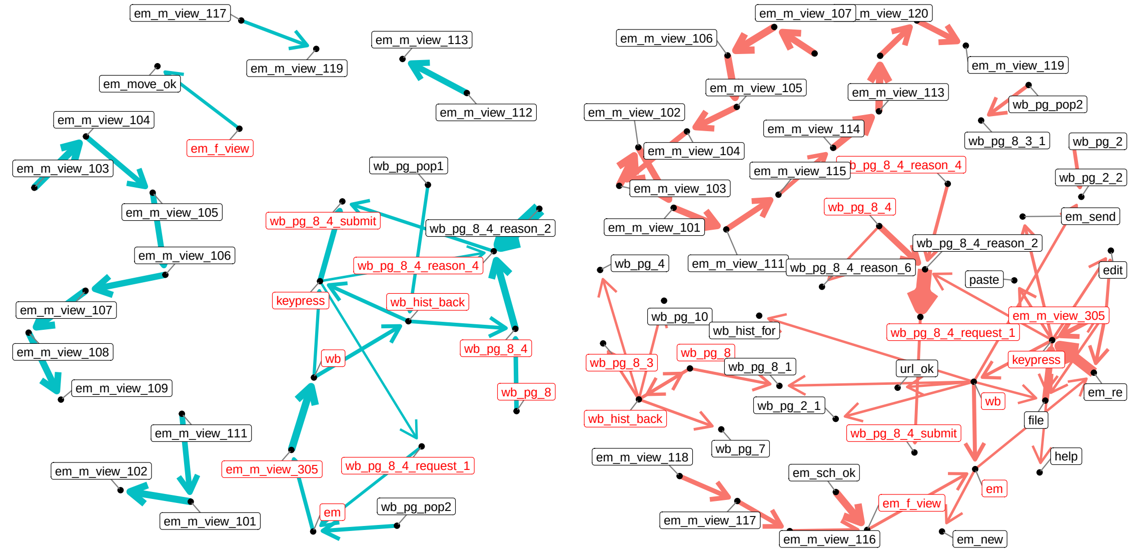

(a) (b)

Figure 24: Network visualization of transition probability differences for the Lamp Return test item in the Norway. (a) shows significantly higher transition probabilities for the correct group (blue arrows), while (b) shows higher probabilities for the incorrect group (red arrows).

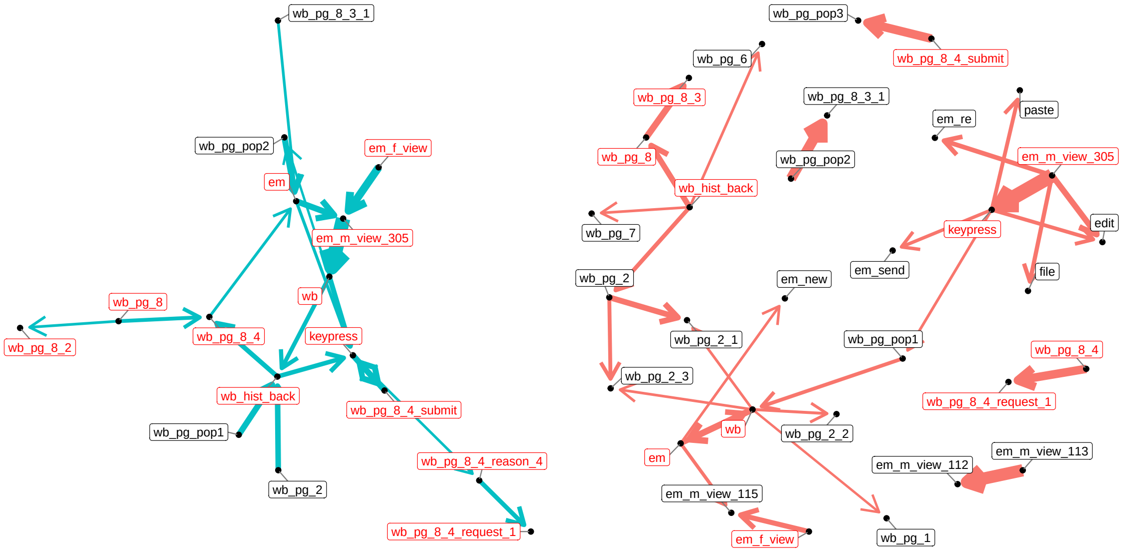

(a) (b)

Figure 25: Network visualization of transition probability differences for the Lamp Return test item in the Poland. (a) shows significantly higher transition probabilities for the correct group (blue arrows), while (b) shows higher probabilities for the incorrect group (red arrows).

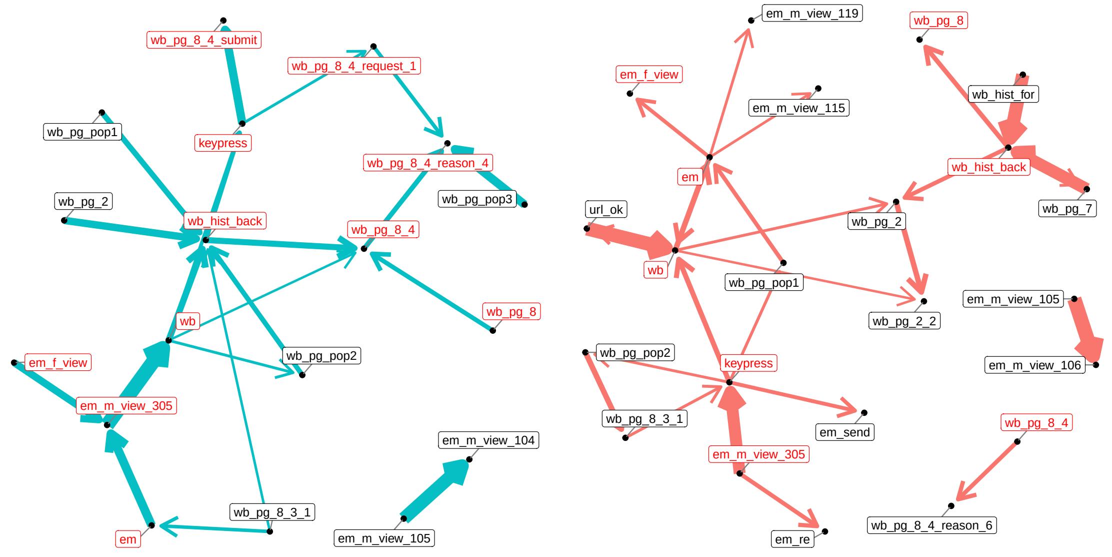

(a) (b)

Figure 26: Network visualization of transition probability differences for the Lamp Return test item in the Slovakia. (a) shows significantly higher transition probabilities for the correct group (blue arrows), while (b) shows higher probabilities for the incorrect group (red arrows).

6 MCMC diagnostic

6.1 Gelman-Rubin's $\hat{R}$ statistics

Figure 27 is the histogram of the Gelman-Rubin's $\hat{R}$ statistics for all parameters in the case of the CD Tally test item for the USA.

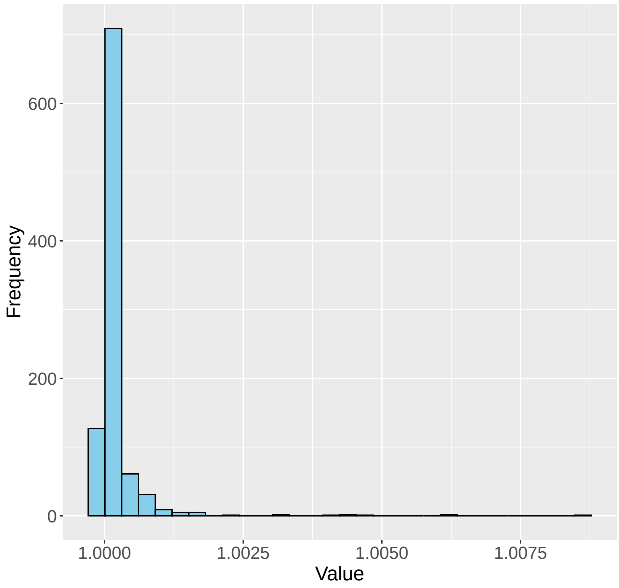

Figure 27: Histogram of the Gelman-Rubin's $\hat{R}$ statistics for all parameters in the case of the CD Tally test item for the USA.

6.2 Trace plots of the MCMC samples

Due to the large number of trace plots, We have uploaded the trace plots for all countries in github page (GitHub Link). In particular, trace plots of five parallel MCMC chains were generated for the USA CD Tally test item to evaluate convergence.

CD Tally test item for USA

The Figure 28 shows trace plots for $\boldsymbol{\alpha}$, $\beta_{0,\cdot}$, and $\beta_{1,\cdot}$. Trace plots for the remaining parameters are available in the GitHub repository.

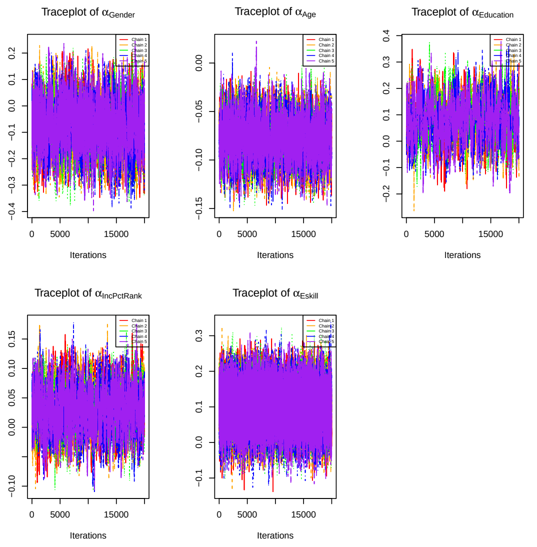

(a) $\boldsymbol{\alpha}$

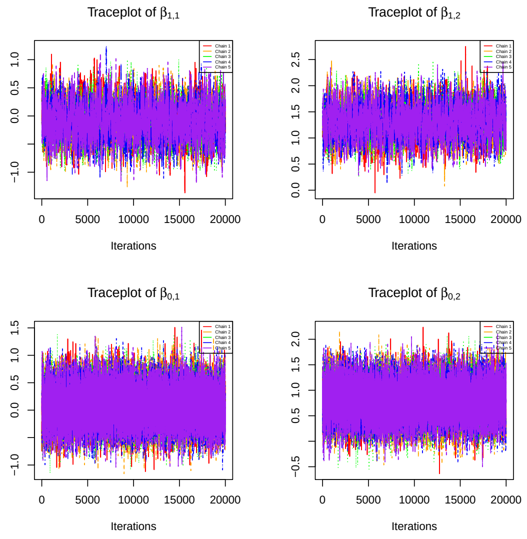

(b) $\boldsymbol{\beta}$

Figure 28: Trace plots of $\boldsymbol{\alpha}$ and β for the Lamp Return test item in the USA, respectively.

7 Sensitivity Analysis

To evaluate the robustness of the proposed model, we conducted a prior sensitivity analysis for key parameters under varying levels of prior informativeness. While results for $\boldsymbol{\beta}$ are presented in the main manuscript, this section reports corresponding analyses for $\boldsymbol{\alpha}$, $\boldsymbol{\kappa}$, and $\boldsymbol{\tau}$.

Parameter	σ_α	Mean	SD	HPD Interval
α_{Gender}	0.5	-0.070	0.090	$[-0.241,\ 0.093]$
	1	-0.070	0.090	$[-0.240,\ 0.088]$
	2	-0.070	0.080	$[-0.231,\ 0.098]$
	5	-0.070	0.080	$[-0.251,\ 0.091]$
	10	-0.070	0.080	$[-0.248,\ 0.095]$
α_{Age}	0.5	-0.080	0.020	$[-0.119,\ -0.040]$
	1	-0.080	0.020	$[-0.116,\ -0.040]$
	2	-0.080	0.020	$[-0.117,\ -0.038]$
	5	-0.080	0.020	$[-0.115,\ -0.038]$
	10	-0.080	0.020	$[-0.118,\ -0.041]$
$\alpha_{\text{Education}}$	0.5	0.080	0.080	$[-0.060,\ 0.241]$
	1	0.090	0.070	$[-0.063,\ 0.228]$
	2	0.080	0.070	$[-0.061,\ 0.226]$
	5	0.090	0.070	$[-0.067,\ 0.226]$
	10	0.080	0.080	$[-0.070,\ 0.227]$
$\alpha_{\text{IncPctRank}}$	0.5	0.030	0.040	$[-0.040,\ 0.106]$
	1	0.030	0.040	$[-0.041,\ 0.095]$
	2	0.030	0.040	$[-0.044,\ 0.102]$
	5	0.030	0.040	$[-0.038,\ 0.099]$
	10	0.030	0.040	$[-0.035,\ 0.099]$
α_{Eskill}	0.5	0.100	0.060	$[-0.008,\ 0.208]$
	1	0.100	0.060	$[-0.010,\ 0.204]$
	2	0.100	0.060	$[-0.008,\ 0.212]$
	5	0.100	0.060	$[-0.012,\ 0.208]$
	10	0.100	0.060	$[-0.012,\ 0.210]$

Table 14: Prior sensitivity analysis results for α. The table summarizes the posterior means, standard deviations, and 95% HPD intervals under different prior standard deviations, σ_α

We examined the sensitivity of posterior estimates for $\boldsymbol{\alpha}$ under normal priors with standard deviations $\sigma_\alpha \in \{0.5, 1, 2, 5, 10\}$. As shown in Table 14, posterior means and standard deviations remained virtually unchanged across prior settings for all covariates, with only minor differences observed in the 95% highest posterior density (HPD) intervals. These results indicate that the estimation of $\boldsymbol{\alpha}$ is highly robust to prior specification, suggesting that the data provide sufficient information to dominate the prior.

For parameters with gamma priors—$\boldsymbol{\kappa}$ (transition-specific) and $\boldsymbol{\tau}$ (individual-specific)—we tested

five (a, b) combinations: (0.001, 1000), (0.01, 100), (0.1, 10), (0.5, 2), and (1, 1), where a and b denote the shape and scale parameters of the Gamma distribution, respectively. Due to the large number of parameters in both cases, we visualized posterior means and corresponding 95% HPD intervals using grouped bar plots. Each bar represents a parameter (i.e., a transition pair or an individual), with color indicating the prior setting, and vertical lines on each bar denoting the 95% HPD interval. Specifically, colors represent Gamma(a, b) configurations as follows: red $=$ (0.001, 1000), orange $=$ (0.01, 100), green $=$ (0.1, 10), blue $=$ (0.5, 2), and purple $=$ (1, 1).

Figures 29 and 30 present the prior sensitivity results for $\boldsymbol{\kappa}_1$ (correct group), while Figures 31 and 32 correspond to $\boldsymbol{\kappa}_0$ (incorrect group). For certain transitions—such as from action 4 to action 5, and from action 5 to action 6—the posterior estimates and 95% HPD intervals under the red bar (Gamma(0.001, 1000)) deviated notably from those under other priors, indicating sensitivity in these specific cases. For most other transitions, differences across prior settings were minor. The main results reported in the manuscript use the Gamma(1, 1) prior for $\boldsymbol{\kappa}$.

Figures 33 through 37 display the results for $\boldsymbol{\tau}$. Across all $\boldsymbol{\tau}$, posterior means and HPD intervals remained stable regardless of the prior specification, suggesting that the estimation of $\boldsymbol{\tau}$ is robust to prior variation. The main results reported in the manuscript use the Gamma(1, 1) prior for $\boldsymbol{\tau}$.

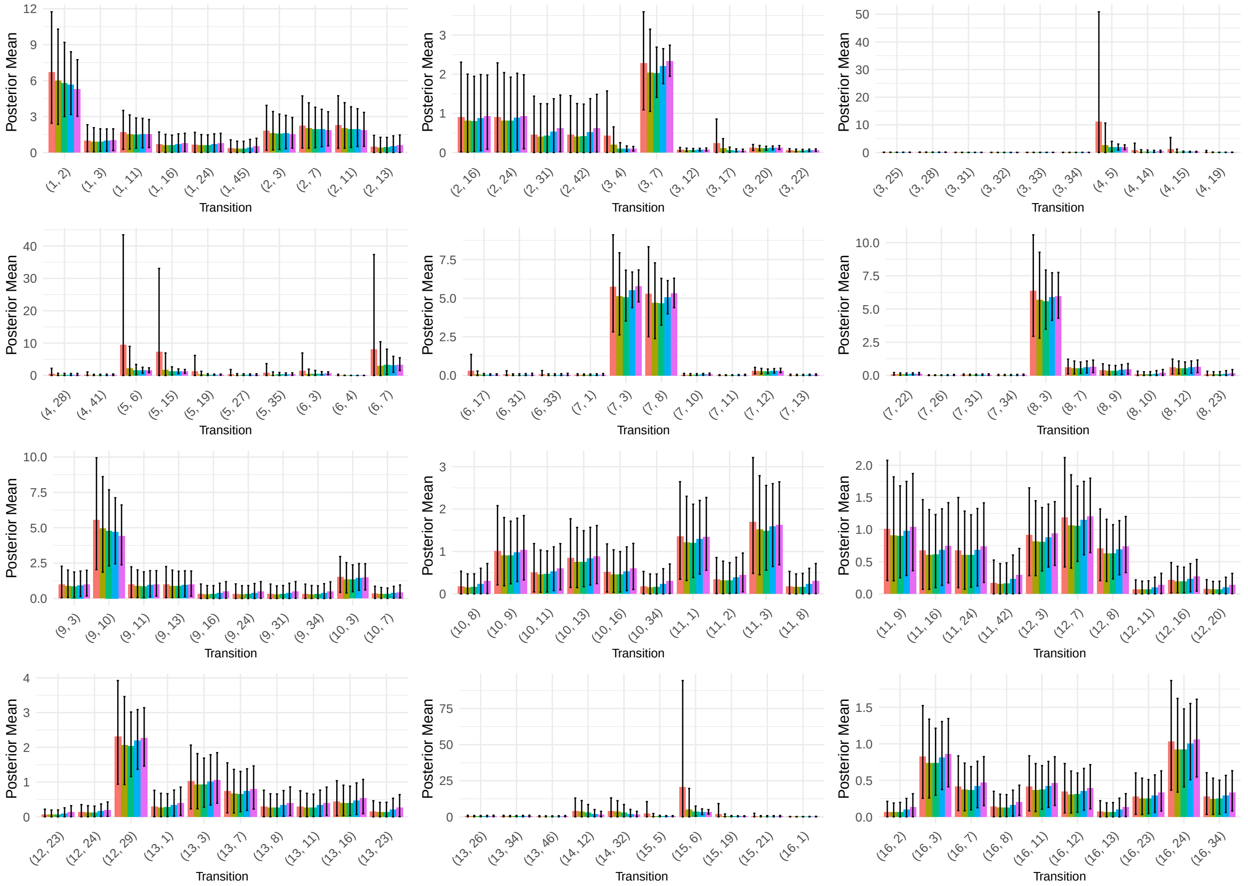

Figure 29: Posterior means and 95% highest posterior density (HPD) intervals for $\boldsymbol{\kappa_1}$ under five gamma prior settings: (0.001, 1000), (0.01, 100), (0.1, 10), (0.5, 2), and (1, 1). Each subplot displays grouped bar plots for 10 transition, with colors indicating prior settings and vertical lines representing 95% HPD intervals. Bars are color-coded by prior setting as follows: red = (0.001, 1000), orange = (0.01, 100), green = (0.1, 10), blue = (0.5, 2), and purple = (1, 1).

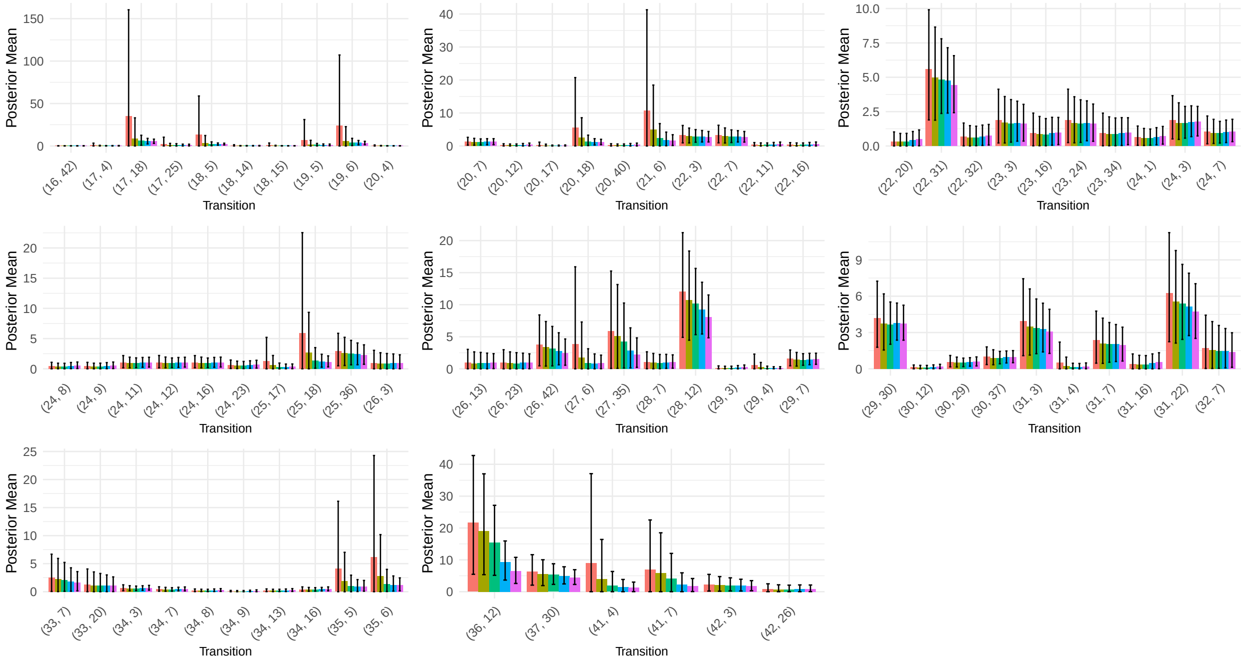

Figure 30: Continuation of Figure 29. Posterior means and 95% highest posterior density (HPD) intervals for $\boldsymbol{\kappa_1}$ under five gamma prior settings: (0.001, 1000), (0.01, 100), (0.1, 10), (0.5, 2), and (1, 1).

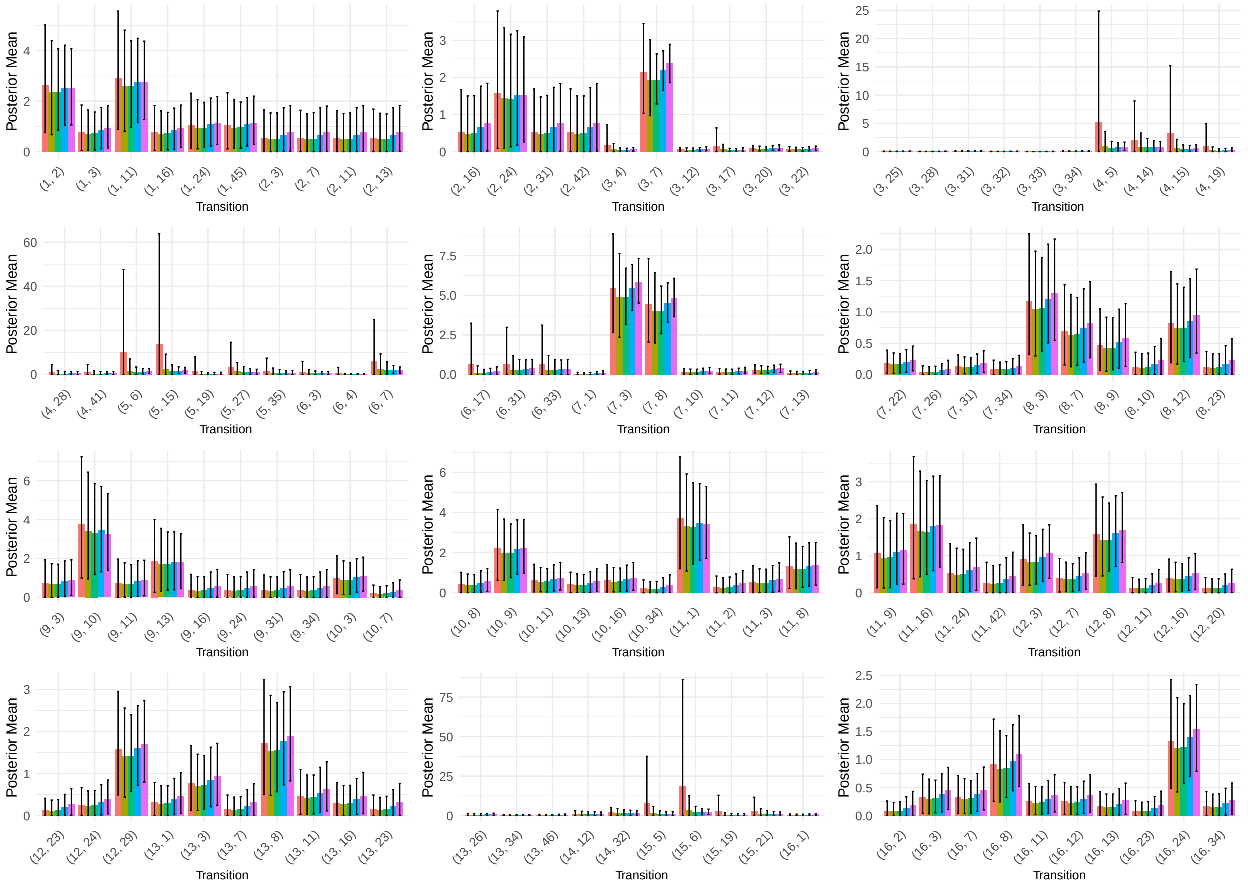

Figure 31: Posterior means and 95% highest posterior density (HPD) intervals for $\boldsymbol{\kappa_0}$ under five gamma prior settings: (0.001, 1000), (0.01, 100), (0.1, 10), (0.5, 2), and (1, 1). Each subplot displays grouped bar plots for 10 transition, with colors indicating prior settings and vertical lines representing 95% HPD intervals. Bars are color-coded by prior setting as follows: red = (0.001, 1000), orange = (0.01, 100), green = (0.1, 10), blue = (0.5, 2), and purple = (1, 1).

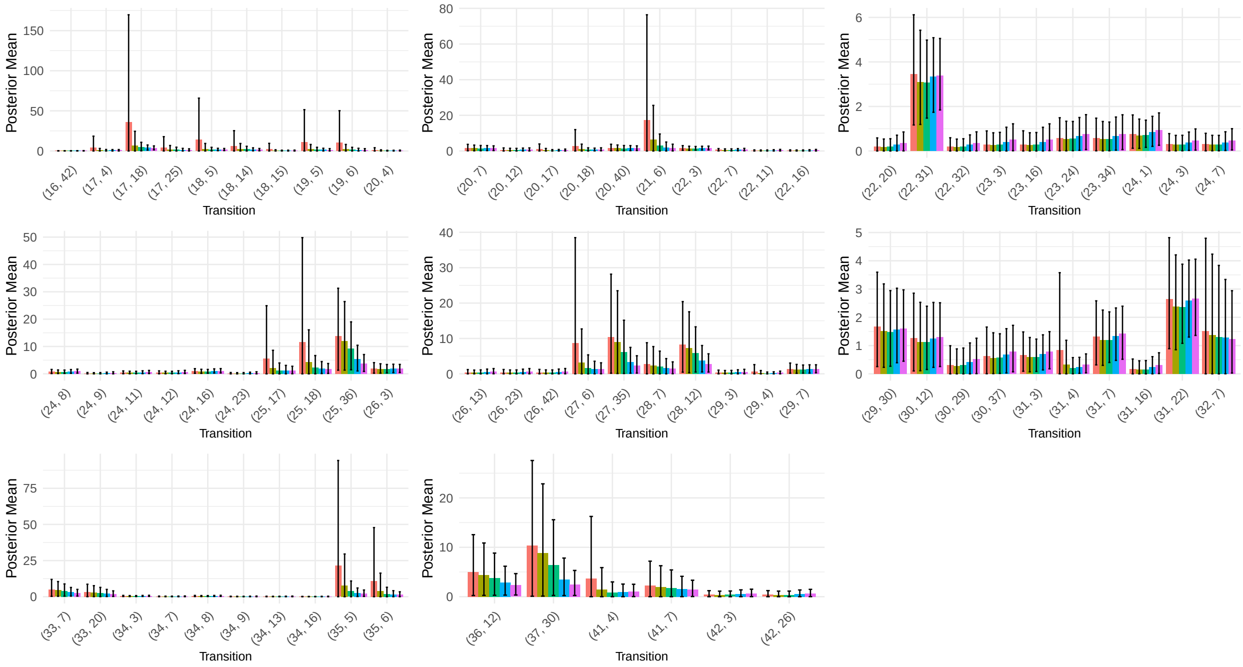

Figure 32: Continuation of Figure 31. Posterior means and 95% highest posterior density (HPD) intervals for $\boldsymbol{\kappa}_0$ under five gamma prior settings: (0.001, 1000), (0.01, 100), (0.1, 10), (0.5, 2), and (1, 1).

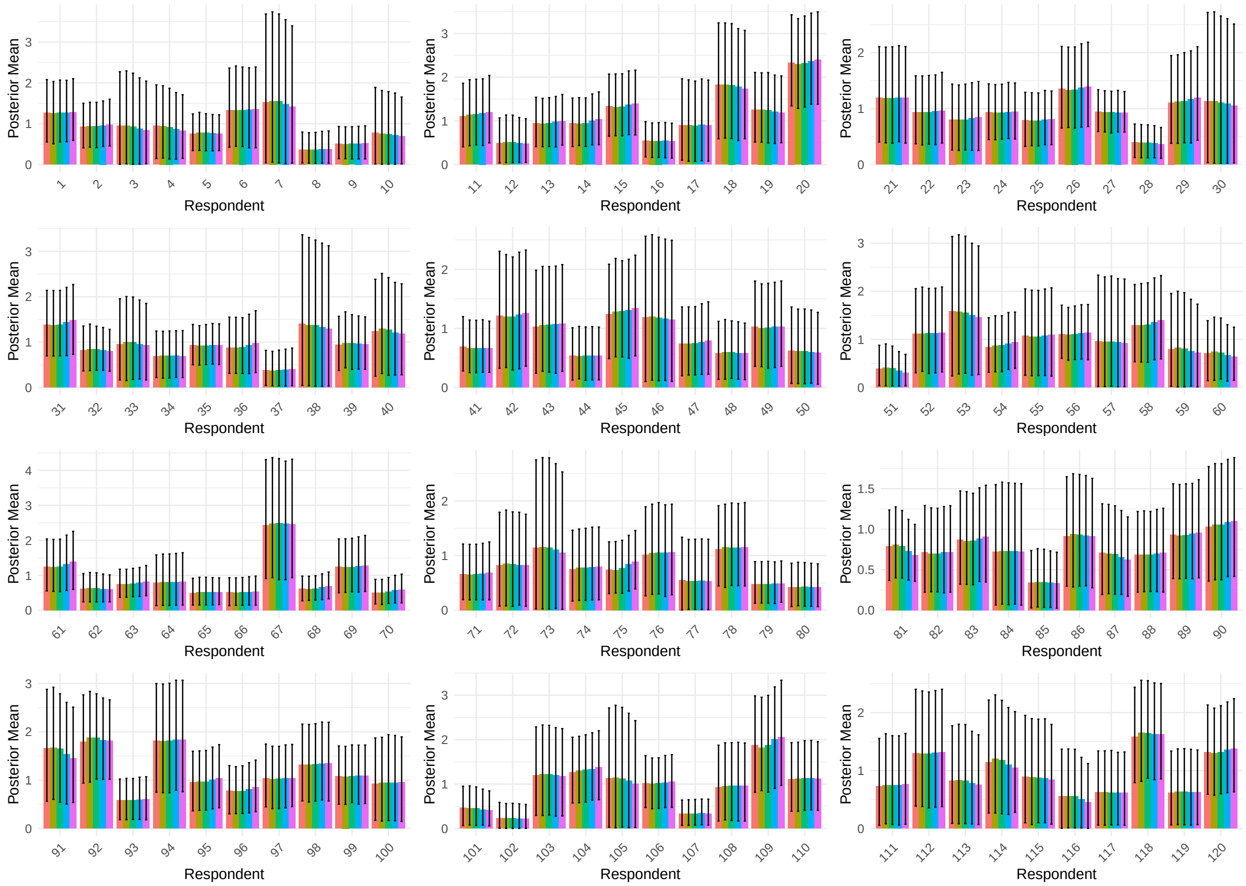

Figure 33: Posterior means and 95% highest posterior density (HPD) intervals for $\boldsymbol{\tau}$ under five gamma prior settings: (0.001, 1000), (0.01, 100), (0.1, 10), (0.5, 2), and (1, 1). Each subplot displays grouped bar plots for 10 individuals, with colors indicating prior settings and vertical lines representing 95% HPD intervals. Bars are color-coded by prior setting as follows: red = (0.001, 1000), orange = (0.01, 100), green = (0.1, 10), blue = (0.5, 2), and purple = (1, 1).

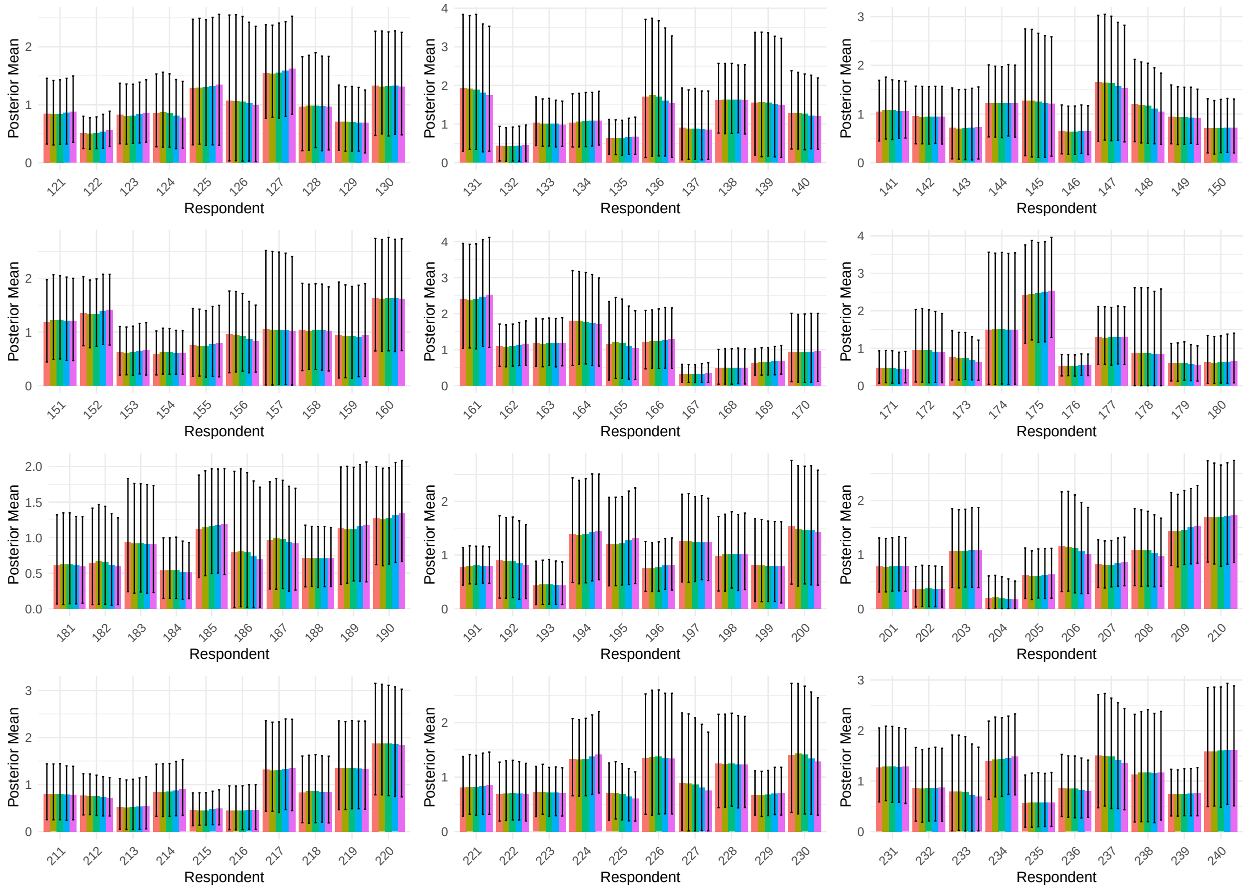

Figure 34: Continuation of Figure 33. Posterior means and 95% highest posterior density (HPD) intervals for $\boldsymbol{\tau}$ under five gamma prior settings: (0.001, 1000), (0.01, 100), (0.1, 10), (0.5, 2), and (1, 1).

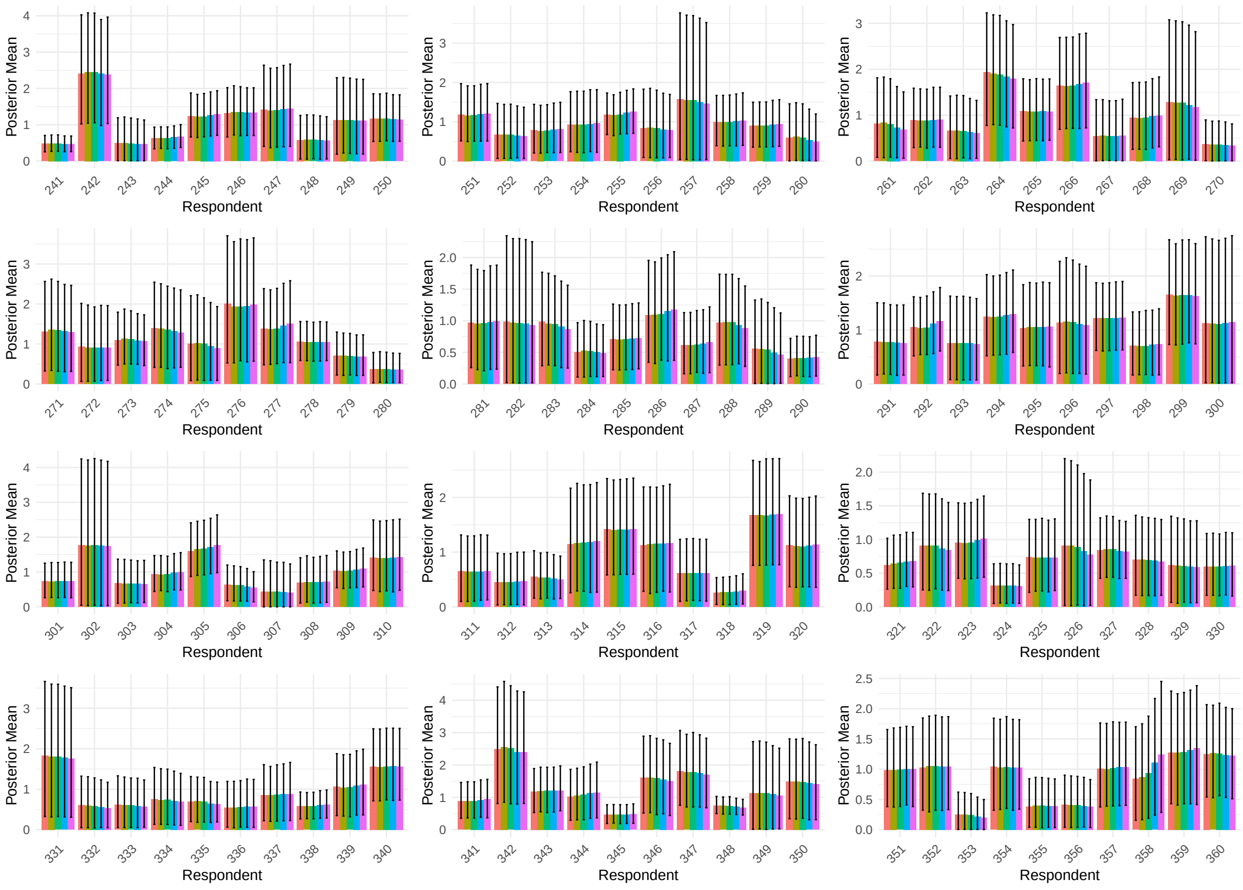

Figure 35: Continuation of Figure 33. Posterior means and 95% highest posterior density (HPD) intervals for $\boldsymbol{\tau}$ under five gamma prior settings: (0.001, 1000), (0.01, 100), (0.1, 10), (0.5, 2), and (1, 1).

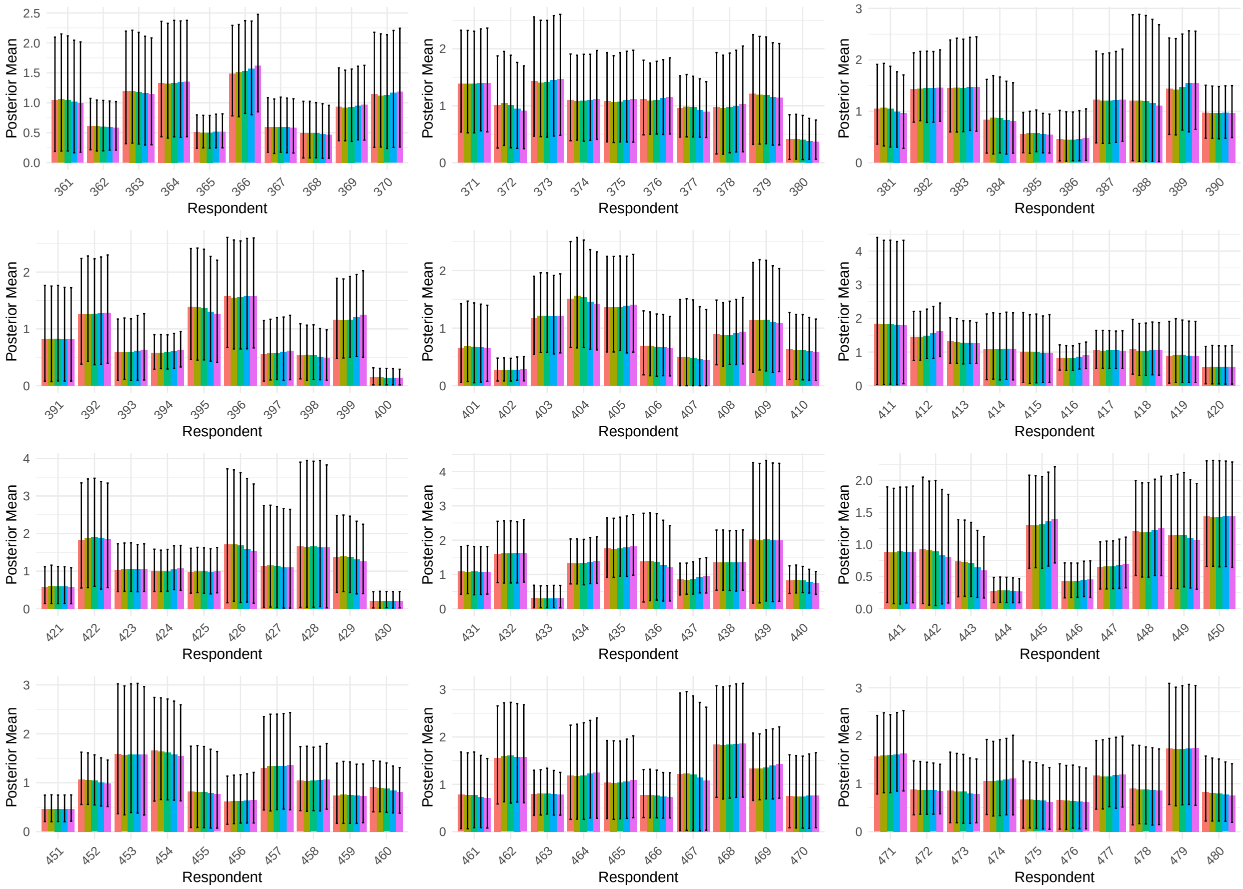

Figure 36: Continuation of Figure 33. Posterior means and 95% highest posterior density (HPD) intervals for $\boldsymbol{\tau}$ under five gamma prior settings: (0.001, 1000), (0.01, 100), (0.1, 10), (0.5, 2), and (1, 1).

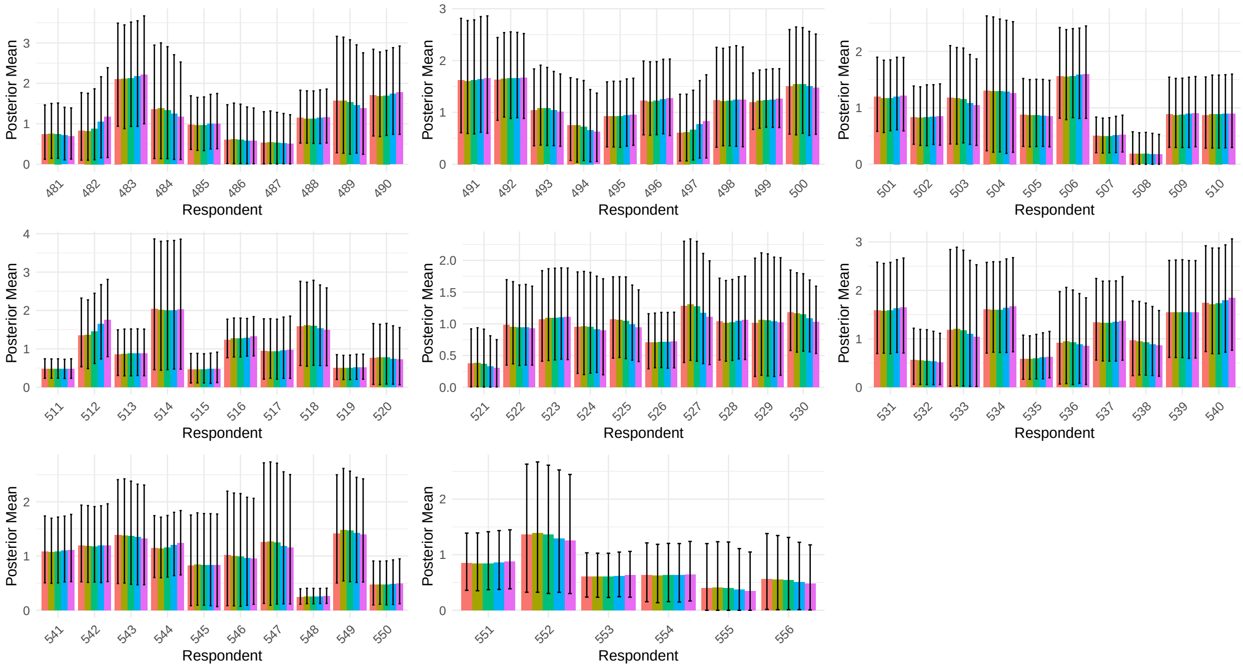

Figure 37: Continuation of Figure 33. Posterior means and 95% highest posterior density (HPD) intervals for $\boldsymbol{\tau}$ under five gamma prior settings: (0.001, 1000), (0.01, 100), (0.1, 10), (0.5, 2), and (1, 1).

8 Simulation Study

This section provides detailed descriptions of the simulation settings used to evaluate the parameter recovery performance of the proposed Multi-State Model (MSM) framework.

General Data Generation Procedure In each scenario, data were generated for 500 individuals. Each individual performed a sequence of actions drawn from a pool of 50 possible actions, including 10 predefined key actions. Five covariates were independently sampled from a normal distribution with mean 0 and variance 4. The number of actions per individual was drawn from a negative binomial distribution with parameters $r = 5$ and $p = 1/3$, capturing over-dispersion commonly observed in real-world log data.

Action sequences were constructed using a competing risks process: at each decision point, transition times were sampled from exponential distributions whose rates were determined by transition-specific hazards $\lambda_{m,l,i}$. The transition with the minimum sampled time was selected as the next action, and the cumulative time was updated accordingly.

Transition Hazard Structure The baseline simulation setting assumed no group-level differences in transition behavior. Transition-specific baseline hazards $\kappa_{g,m,l}$ (for group $g = 0, 1$) were drawn from the following Gamma distribution:

$$\kappa_{g,m,l} \sim \text{Gamma}\left(\exp\left(\beta_{g,1} \cdot \mathbb{I}_{\{m \in \text{key}\}} + \beta_{g,2} \cdot \mathbb{I}_{\{l \in \text{key}\}}\right),\ 0.01\right)$$

where $\mathbb{I}.$ is an indicator function that returns 1 if the condition is true. This structure allowed key action effects to influence the transition intensity based on the status of the start and end actions. The parameter τ_i were independently drawn from a Gamma(1, 0.01) distribution.

Scenarios Setting We designed four simulation scenarios to examine model performance under varying effects of key actions and covariates.

- **Scenario 1: Baseline (No Group Differences)**

  All individuals shared identical transition probabilities. Key action effects, $(\beta_{0,1}, \beta_{0,2}, \beta_{1,1}, \beta_{1,2})$, were all fixed at $(3, 3, 3, 3)$, and covariate effects α_p $(p = 1, 2, 3, 4, 5)$ were set to 1 for all individuals.

- **Scenario 2: Heterogeneous Covariate Effects**

  Baseline hazards and key action effects remained the same as in Scenario 1, but covariate effects α_p ($p = 1, 2, 3, 4, 5$) were set to $(-2, 1, 0, 1, 2)$ to introduce covariate-level heterogeneity.

- **Scenario 3: Group Differences in Key Action Effects (Start and End)**

  Key action effects varied by group: for group 0, $(\beta_{0,1}, \beta_{0,2}) = (3, 3)$; for group 1, $(\beta_{1,1}, \beta_{1,2}) = (-1, -1)$, introducing opposing transition tendencies at both the origin and destination actions.

- **Scenario 4: Group Differences in Key Action Effects (One Side Only)**

  Same as Scenario 3, except group 1 had asymmetric key action effects: $(\beta_{1,1}, \beta_{1,2}) = (3, -3)$.

The simulation results are summarized and discussed in the main manuscript.
